# Physical behaviors of 12-15 year-old adolescents in 54 low- and middle-income countries: Results from the Global School-based Student Health Survey

**DOI:** 10.7189/jogh.10.010423

**Published:** 2020-06

**Authors:** Guodong Xu, Ning Sun, Lian Li, Wanfu Qi, Changwei Li, Maigeng Zhou, Zhu Chen, Liyuan Han

**Affiliations:** 1 Hwa Mei Hospital, University of Chinese Academy of Sciences, Ningbo, Zhejiang, PR China; 2 Ningbo Institute of Life and Health Industry, University of Chinese Academy of Sciences, Ningbo, Zhejiang, PR China; 3 Ningbo Medical Center Lihuili Hospital, Ningbo, Zhejiang, PR China; 4 Ningbo College of Health Sciences, Ningbo, Zhejiang, PR China; 5 Zhejiang Provincial Key Laboratory of Pathophysiology, School of Medicine, Ningbo University, Ningbo, PR China; 6 Ningbo Health Supervision Institute, Ningbo, Zhejiang, PR China; 7 Department of Epidemiology and Biostatistics, University of Georgia College of Public Health, Athens, Georgia, USA; 8 National Center for Chronic and Noncommunicable Disease Control and Prevention, Chinese Center for Disease Control and Prevention, Beijing, PR China

## Abstract

**Background:**

To describe and compare the separate and combined prevalence of physical activity, active transportation, physical education, and sedentary behavior among adolescents 12-15 year-olds in low- and middle-income countries (LMICs).

**Methods:**

We used the latest data from the Global School-based Student Health Survey (GSHS), which collect data on the physical behaviors of young adolescents in LMICs. The weighted prevalence and 95% confidence intervals of separate, combined and all of the qualifying physical behaviors were calculated. Pooled overall and regional estimates were calculated using a random effects model.

**Results:**

In total, 154 559 young adolescents (45.90% boys) aged 12-15 from 54 countries covered in the GSHS were included in our analysis. Only 0.7% (95% confidence interval (CI) = 0.5%-1.0%) of the adolescents, comprising 0.9% (95% CI = 0.6%-1.3%) of the boys and 0.5% (95% CI = 0.3%-0.7%) of the girls, displayed all of the qualifying physical behaviors. The overall prevalence of physical activity, active transportation, physical education, and sedentary behavior was 15.2% (95% CI = 13.7%-16.7%), 39.5% (95% CI = 34.9%-44.0%), 18.8% (95% CI = 16.1%-21.5%), and 34.6% (95% CI = 28.4%-40.7%), respectively. The overall prevalence of high levels of combined physical behaviors was 6.6% (95% CI = 5.4%-7.8%), with lowest in the Eastern Mediterranean region (4.9%, 95% CI = 3.5%-6.2%) and highest in Southeast Asia (8.6%, 95% CI = 4.9%-12.3%).

**Conclusion:**

The prevalence of the separate physical behaviors and high levels of the combined physical behaviors was consistently low among young adolescents in LMICs, and that of all qualifying physical behaviors was even lower.

Globally, physical inactivity and sedentary behavior are highly prevalent in adolescents [1]. Reportedly, only approximately 23.8% of boys and 15.4% of girls engage in at least 60-minute of physical activity per day [2]. In addition, more than half of the adolescents sit for >3 hours per day outside school [3].

Accumulating evidence has suggested that physical activity has protective effects against cardiovascular diseases [4], diabetes [5], and cancer [6], whereas sedentary behavior increases the risk of obesity and cardiometabolic diseases [7,8]. However, the risk attributable to sedentary behavior depends on the amount of moderate-to-vigorous physical activity [9,10]. More than 1 hour of moderate-intensity physical activity can eliminate the increased risk of death associated with a long sitting time [11]. However, if the sitting time is more than 5 hours per day, mortality significantly increases regardless of the intensity of physical activity [11].

Notably, engaging in active transportation and attending physical education increase the chances of meeting physical activity recommendations among adolescents [12,13]. Active commuting to and from school can help in increasing daily physical active levels in youth [14], and approximately 50% of physical education lesson time is spent on physical activity [15]. Taken together, these findings suggest that physical activity, active transportation, physical education, and sedentary behavior together influence adolescent health.

A study described worldwide physical activity levels, showing 80% of adolescents do not reach recommended levels of physical activity, and more than 65% spend 2 hours or more per day watching television from the data of 41 countries in Europe and North America [1]. However, most studies have focused the prevalence of physical activity, active transportation, physical education, and sedentary behavior in adolescents separately [3]. To date, no study has estimated the combined prevalence of the four above mentioned physical behaviors in young adolescents in low- and middle-income countries (LMICs). Therefore, using the most current GSHS data sets of 2009-2015, we described the separate and combined prevalence of physical activity, active transportation, physical education, and sedentary behavior, as well as all qualifying physical behaviors, in 12-15 year-old adolescents in 54 LMICs and examined the differences between subgroups stratified by gender, age, and body mass index (BMI).

## MATERIALS and METHODS

### Data sources

The detailed methods used in the GSHS have been described online on the World Health Organization (WHO) website (http://www.who.int/ncds/surveillance/gshs/en/), on the Centers for Disease Control and Prevention (CDC) website (https://www.cdc.gov/gshs/index.htm), and in previous reports [16,17]. The GSHS is a multicenter, multiethnic, school-based, and ongoing collaborative surveillance project led by WHO and the United States CDC, mainly conducted to assess health behaviors and protective factors among 12-15 year-old adolescents. The GSHS uses a self-administered questionnaire to obtain data on adolescent health behaviors and protective factors in 10 key areas, namely alcohol use, dietary behaviors, drug use, hygiene, mental health, physical activity, protective factors, sexual behaviors, tobacco use, and violence and unintentional injury. The data are collected during regular school hours using a self-report computer-scannable form. The questionnaire is translated into the appropriate language for each country, if necessary. All responses are anonymous, and no identifiable information is collected. Countries can select the sections of the questionnaire they wish to use. However, the questions within the chosen survey sections must be used without modification, so the results are directly comparable across countries and over repeat assessments [16,17].

A standardized two-stage random cluster sampling procedure was used to select eligible participants from each country [18]. Approval for the GSHS was obtained from the local national government administrations of all countries. Participation by adolescents in all countries was voluntary, and written consent was obtained from them or their parents.

### Definitions of physical activity, active transportation, physical education, and sedentary behavior

For physical activity, the form included the following question: “During the past 7 days, on how many days were you physically active for a total of at least 60 min per day?” The response options were “0 days,” “1 day,” “2 days,” “3 days,” “4 days,” “5 days,” “6 days,” or “7 days.” Adolescents who were physically active for at least 1 hour per day were considered to engage in “physical activity” [3].

For active transportation, the form included the following question: “During the last 7 days, on how many days did you walk or ride a bicycle to or from school?” The response options were the same as above. Adolescents who walked or rode a bicycle to or from school for at least 3 days per week were considered to engage in “active transportation” [3].

For physical education, the form included the following question: “During this school year, on how many days did you go to a physical education class each week?” The response options were “0 days,” “1 day,” “2 days,” “3 days,” “4 days,” or “5 or more days.” Adolescents who went to physical education class at least 5 days per week were considered to attend “physical education” [3].

For sedentary behavior, the form included the following question: “How much time do you spend during a typical or usual day sitting and watching television, playing computer games, talking with friends, or doing other seated activities such as surfing the Internet?” The response options were “less than 1 h per day,” “1 to 2 h per day,” “3 to 4 h per day,” “5 to 6 h per day,” “7 to 8 h per day,” or “more than 8 h per day.” Adolescents who spent 3 or more hours per day sitting down were classified as having “sedentary behavior” [3].

### All qualifying and combined physical behaviors

Adolescents who were physically active, engaged in active transportation, attended physical education classes, and had no sedentary behavior were classified as having all qualifying physical behaviors.

For physical activity and active transportation in the past 7 days, we assigned scores from 0 to 7 for 0 to 7 days, respectively. For physical education in the past 7 days, we assigned scores from 0 to 4 for 0 to 4 days, respectively, and 5 for “5 or more days.” For sedentary behavior, we assigned scores of 5 for “less than 1 h per day,” 4 for “1 to 2 h per day,” 3 for “3 to 4 h per day,” 2 for “5 to 6 h per day,” 1 for “7 to 8 h per day,” and 0 for “more than 8 h per day.”

The combined physical behaviors of the young adolescents were calculated by summing the scores of the four above mentioned survey items, with the highest possible scores being 24. A higher score indicated better physical behavior. In addition, the combined physical behaviors of young adolescents were grouped into the following quartiles: low (score 0-6), moderately low (score 7-12), moderately high (score 13-18), or high (score 19-24).

### Statistical analysis

As the GSHS uses a complex sampling design, data analyses should take into account sampling design. The prevalence of physical activity, active transportation, physical education, and sedentary behavior separately, as well as the prevalence of combined physical behaviors, was reported as weighted prevalence values and 95% confidence intervals (CIs) using the SAS SURVEYMEANS procedure. This procedure produces survey population estimates, such as proportions and 95% confidence intervals (CI), from sample survey data while accounting for the sample design used to select the survey sample. In our analyses, we added weights, stratum, and a primary sampling unit (PSU) to every student record in the GSHS data file to reflect the weighting process and the two-stage sampling design. The weights allow the results to be generalized to the study population and the national student population. The stratum reflects the first stage of the GSHS sampling (school level), and the PSU reflects the second stage (classroom level).

Pooled overall and regional estimates were calculated by meta-analysis using the random-effects model. Heterogeneity was assessed using the *I*^2^ statistic. Subgroup analyses were stratified by gender (boys vs girls), age (12-13 years vs 14-15 years), and BMI (normal, overweight, and obesity). The differences in prevalence between gender, age, and BMI groups were estimated using the χ^2^ test. Standard errors were estimated using the Taylor series linearization method [19]. *P* values <0.05 were considered to indicate statistically significant differences.

BMI was calculated as kilograms per square meter (kg/m^2^). The BMI of adolescents was categorized as underweight (<5^th^ percentile), normal (5^th^ to <85^th^ percentiles), overweight (85th to <95th percentiles), and obese (≥95^th^ percentile) based on their age and gender [20]. SAS version 9.4 (SAS Institute, Cary, NC) and STATA version 12.0 (Stata Corporation; College Station, TX, USA) were used to perform statistical analyses.

## RESULTS

**Figure 1:**
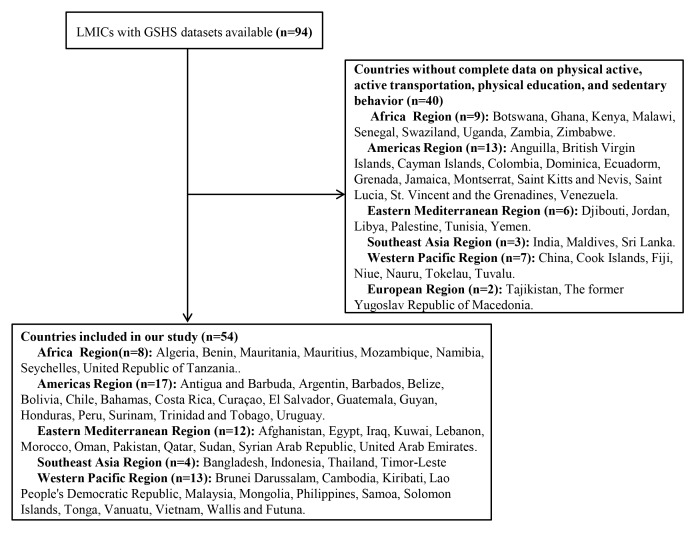
Selection process for low- and middle-income countries (LMICs) using the Global School-based Student Health Survey (GSHS) national data.

**Table 1** summarizes the characteristics of the survey and participants from the GSHS. A total of 154 559 adolescents (45.90% of whom were boys) aged 12-15 years with complete data on physical activity, active transportation, physical education, and sedentary behavior between 2009 and 2015 were included in our study. Fifty-four countries from five WHO regions (8 from Africa, 17 from America, 12 from Eastern Mediterranean, 4 from Southeast Asia, and 13 from Western Pacific) were included (**Figure 1**). Sample sizes ranged from 697 in Mozambique (the Africa region) to 21 762 in Argentina (the America region). The overall response rate was 98.56% (95% CI = 88.39%-99.83%).

**Table 1:** Survey characteristics of the Global School-based Student Health Surveys according to country, 2009-2015

	Survey year	Response rate (%)	Sample size	Boys (%)
**Africa Region:**				
Algeria	2011	99.01	3527	45.51
Benin	2009	99.58	1179	63.60
Mauritania	2010	95.68	1320	45.8
Mauritius	2011	98.03	2081	45.68
Mozambique	2015	97.42	697	50.24
Namibia	2013	97.98	1981	41.51
Seychelles	2015	94.47	2063	44.84
United Republic of Tanzania	2014	96.97	2643	44.68
**Region of the Americas:**				
Antigua and Barbuda	2009	94.05	1243	46.39
Argentina	2012	96.29	21 762	46.38
Barbados	2011	97.88	1507	45.11
Belize	2009	97.10	1621	46.64
Bolivia	2012	98.11	2956	49.76
Chile	2013	96.23	1354	48.43
Bahamas	2013	94.74	1312	44.99
Costa Rica	2009	99.25	2277	47.58
Curaçao	2015	92.51	1508	45.67
El Salvador	2013	98.42	1644	52.72
Guatemala	2015	93.02	3666	47.33
Guyana	2010	98.19	1985	43.61
Honduras	2012	97.60	1502	47.30
Peru	2010	99.83	2374	48.51
Suriname	2009	96.89	1062	46.17
Trinidad and Tobago	2011	95.76	2383	54.39
Uruguay	2012	98.28	2905	46.56
**Eastern Mediterranean Region:**				
Afghanistan	2014	96.14	1530	37.31
Egypt	2011	98.56	2424	45.65
Iraq	2012	95.81	1553	54.79
Kuwait	2015	91.77	2066	46.78
Lebanon	2011	95.79	1995	46.93
Morocco	2010	98.98	2451	50.15
Oman	2015	99.29	1681	43.54
Pakistan	2009	99.10	5005	74.80
Qatar	2011	90.18	1802	44.41
Sudan	2012	96.41	1476	35.87
Syrian Arab Republic	2010	99.29	2941	40.04
United Arab Emirates	2010	96.20	2313	38.28
**Southeast Asia Region:**				
Bangladesh	2014	96.49	2760	38.48
Indonesia	2015	98.02	8824	46.37
Thailand	2015	97.78	4149	46.56
Timor-Leste	2015	98.48	1705	41.67
**Western Pacific Region:**				
Brunei Darussalam	2014	98.41	1827	46.05
Cambodia	2013	98.90	1820	43.76
Kiribati	2011	99.12	1358	41.29
Lao People’s Democratic Republic	2015	99.82	1664	41.98
Malaysia	2012	99.61	16 287	51.15
Mongolia	2013	99.54	3720	47.62
Philippines	2015	98.95	6167	43.37
Samoa	2011	88.39	2213	39.52
Solomon Islands	2011	97.65	979	50.11
Tonga	2010	99.13	1958	44.58
Vanuatu	2011	99.31	865	40.81
Vietnam	2013	99.60	1749	46.79
Wallis and Futuna	2015	92.83	725	48.28
**Total**		**98.56**	**154 559**	**45.90**

**Figure 2:**
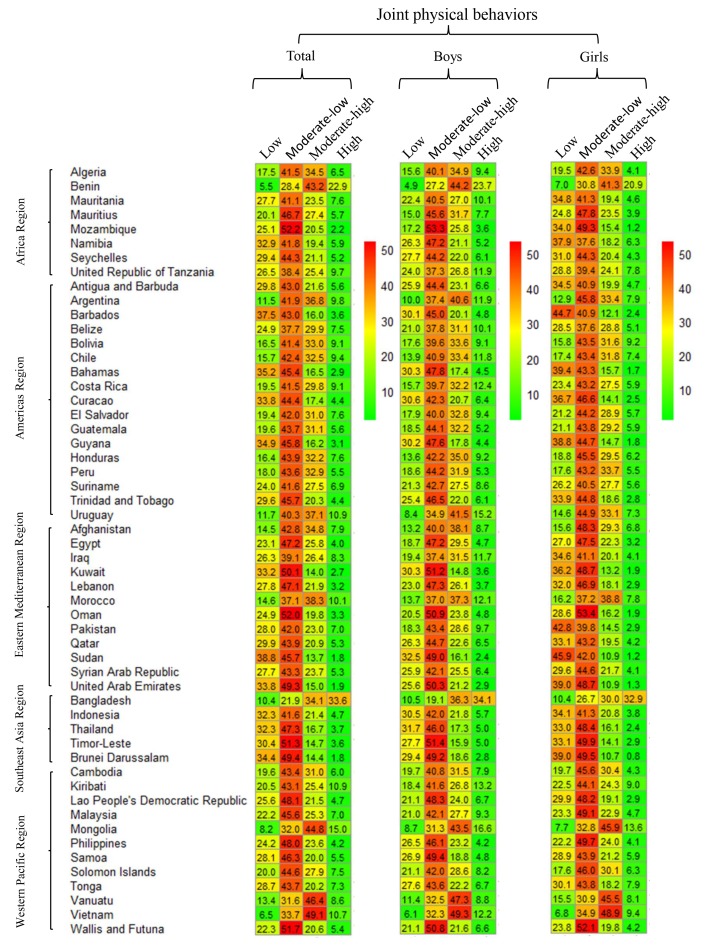
Combined physical behaviors in adolescents aged 12-15 years by gender and country group.

The overall prevalence of low (0-6), moderately low (7-12), and moderately high levels of combined physical behaviors across the 54 countries was 24.1% (95% CI = 21.6%-26.5%), 43.0% (95% CI = 42.1%-43.9%), and 25.3% (95% CI = 22.8%-27.7%), respectively (**Figure 2** and Table S1 in the **Online Supplementary Document**). The overall prevalence of high levels (score 19-24) of combined physical behaviors was 6.6% (95% CI = 5.4%-7.8%), with the lowest in Sudan and Brunei Darussalam (1.8%, 95% CI = 0.9%-2.7%) and 1.8%, 95% CI = 1.1%-2.4%) and the highest in Bangladesh (33.6%, 95% CI = 27.2%-40.0%) (**Figure 2** and Table S1 in the **Online Supplementary Document**).

**Figure 3:**
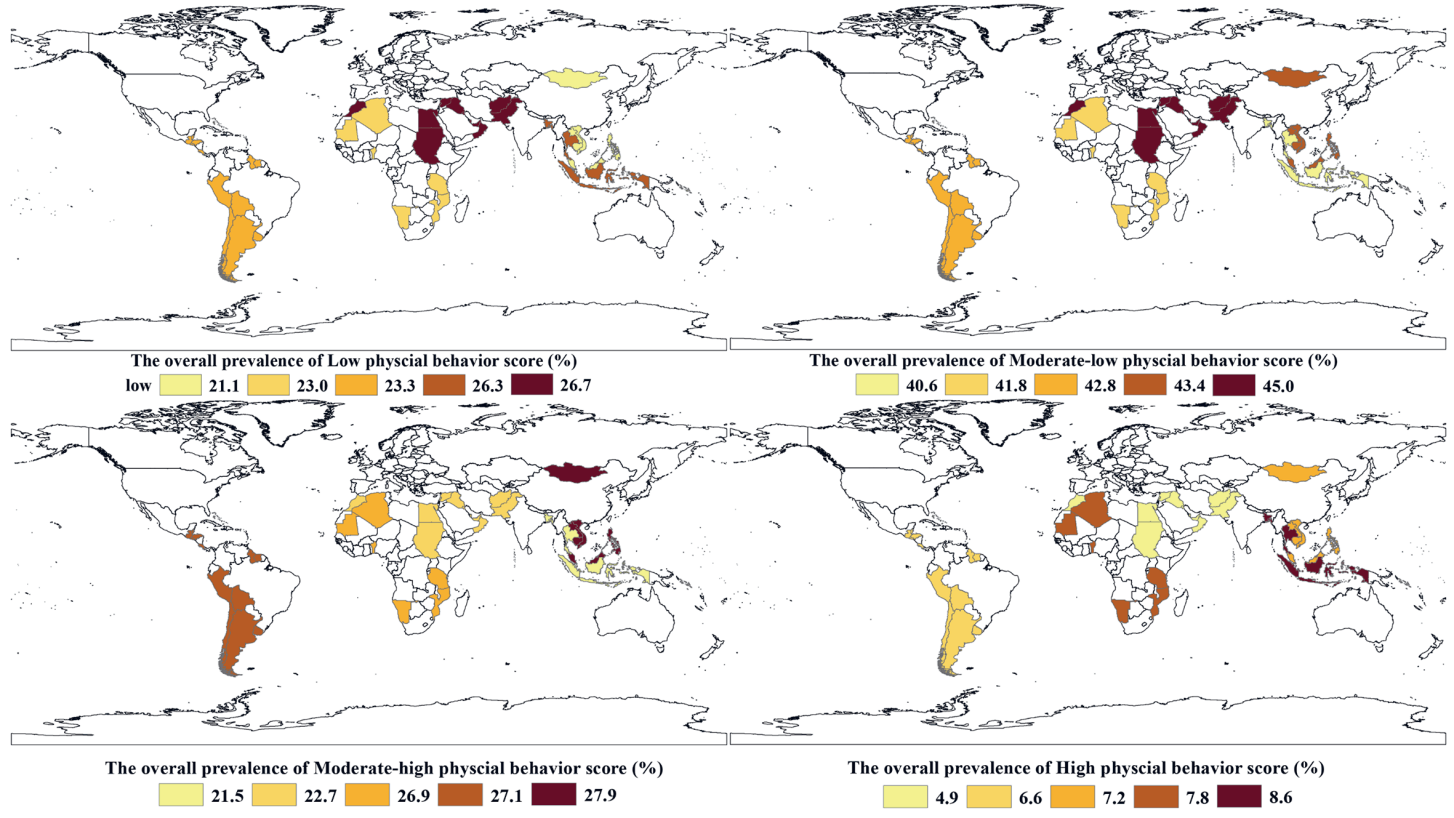
Pooled estimates of combined physical behaviors among adolescents aged 12-15 years by WHO region, 2009-2015.

The pooled prevalence of high levels of combined physical behaviors was the lowest in the Eastern Mediterranean (4.9%. 95% CI = 3.5%-6.2%) and the highest in Southeast Asia (8.6%, 95% CI = 4.9%-12.3%) (**Figure 3** and Table S1 in the **Online Supplementary Document**). The prevalence of high levels of combined physical behaviors was 7.8% (6.5%-9.0%) in boys and >5.1% (3.8%-6.4%) in girls (*P* = 0.02, Table S1 in the **Online Supplementary Document**). No statistical significances were observed between different age or BMI groups (Table S2 and S3 in the **Online Supplementary Document**, respectively).

**Figure 4:**
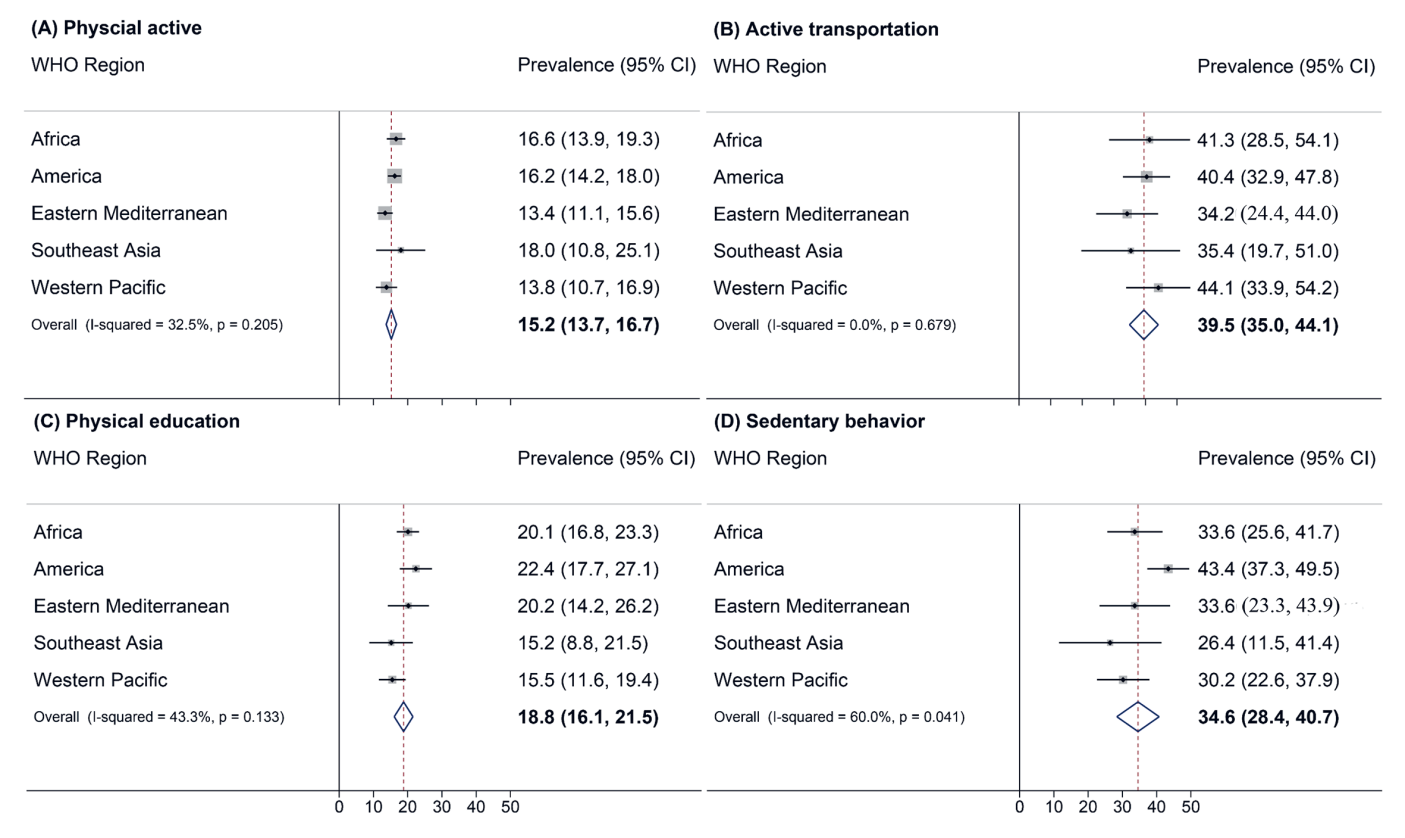
Pooled estimates of percentage of physical active, active transportation, physical education, and sedentary behavior among adolescents aged 12-15 years by WHO region, 2009-2015.

The prevalence of physical activity among young adolescents across the 54 countries was 15.2% (13.7%-16.7%), with the lowest in Cambodia (6.3%, 95% CI = 4.5%-8.2%) and the highest in Bangladesh (42.2%, 95% CI = 37.0%-47.4%) (Table S4 in the **Online Supplementary Document**). Its pooled prevalence was the lowest in the Eastern Mediterranean (13.4%, 95% CI = 11.1%-15.6%) and the highest in Southeast Asia (18.0%, 95% CI = 10.8%-25.1%) (**Figure 4** and Table S4 in the **Online Supplementary Document**). In 40 of the 54 countries (74.1%), the prevalence of physical activity was higher among boys than among girls (all *P* < 0.05), and the pooled prevalence was also higher among boys than among girls (18.7%, 95% CI = 16.6%-20.9%, vs 11.7%, 95% CI = 10.8%-12.7%; *P* = 0.02).

The prevalence of engaging in active transportation among young adolescents across the 54 countries was 39.5% (34.9%-44.0%), with the lowest in the United Arab Emirates (10.4%, 95% CI = 7.3%-13.5%) and the highest in Vietnam (78.8%, 95% CI = 74.5%-82.8%) (Table S5 in the **Online Supplementary Document**). Its pooled prevalence was the lowest in the Eastern Mediterranean (34.2%, 95% CI = 24.4%-44.0%) and the highest in the Western Pacific (44.1% (33.9%-54.2%) (**Figure 4** and Table S5 in the **Online Supplementary Document**). In 20 of the 54 countries, the prevalence of engaging in active transportation was higher in boys than in girls (Table S5 in the **Online Supplementary Document**).

The prevalence of attending physical education classes among young adolescents across the 54 countries was 18.8% (16.1%-21.5%), with the lowest in Peru (1.7%, 95% CI = 0%-3.4) and the highest in Oman (42.7%, 95% CI = 39.4%-46.1%) (Table S6 in the **Online Supplementary Document**). Its pooled prevalence was the lowest in Southeast Asia (15.2%, 95% CI = 8.8%-21.5%) and the highest in America (22.4%, 95% CI = 17.7%-27.1%) (**Figure 4** and Table S6 in the **Online Supplementary Document**). In 15 of the 54 countries, boys and 14-15-year-old adolescents spent more time on physical education than girls and 12-13-year-old adolescents (*P* = 0.01 and 0.02, respectively) (Table S6 in the **Online Supplementary Document**).

The prevalence of sedentary behavior among young adolescents across the 54 countries was 34.6% (28.4%-40.7%), with the lowest in Pakistan (8.3%, 95% CI = 6.6%-9.9%) and the highest in Barbados (65.2%, 95% CI = 62.1%-68.3%) (Table S7 in the **Online Supplementary Document**). Its pooled prevalence was the lowest in Southeast Asia (26.4%, 95% CI = 11.5%-41.4%) and the highest in America (43.4%, 95% CI = 37.3%-49.5%) (**Figure 4** and Table S7 in the **Online Supplementary Document**). In 20 of the 54 countries, the prevalence of sedentary behavior was higher in 14-15-year-old adolescents than in younger adolescents (Table S7 in the **Online Supplementary Document**). The pooled prevalence of sedentary behavior was also higher in 14-15-year-old adolescents than in 12-13-year-old adolescents (36.5%, 95% CI = 30.0%-43.0%, vs 31.3%, 95% CI = 25.5%-37.7%; *P* = 0.01).

Only 0.7% (95% CI = 0.5%-1.0%) of young adolescents across the 54 countries displayed all qualifying physical behaviors, with the lowest in Cambodia (0.02%, 95% CI = 0.0%-0.1%) and the highest in Uruguay (2.4%, 95% CI = 1.6%-3.2%) (Table S8 in the **Online Supplementary Document**). The pooled prevalence was the lowest in Southeast Asia (0.4%, 95% CI = 0.2%-0.7%) and the highest in America (1.1%, 95% CI = 0.8%-1.4%) (Table S8 in the **Online Supplementary Document**). The pooled prevalence was higher among boys than among girls (0.9%, 95% CI = 0.6%-1.3%, vs 0.5%, 95% CI = 0.3%-0.7%; *P* = 0.03).

## DISCUSSION

Our survey summarized the latest GSHS data (from 2009 to 2015) on physical activity, active transportation, physical education, and sedentary behavior among 12-15 year-old adolescents in 54 LMICs. The prevalence of separate and high levels of combined physical behaviors was consistently low among young adolescents in LMICs, and that of all of the qualifying physical behaviors was even lower. The same questions for the assessment of physical activity were taken in 41 Europe and North America countries, and the results from 2005/2006 survey also showed the low prevalence of sufficient physical activity (25% and 15% in 13-year-olds boys and girls, respectively) [21].

The overall prevalence of high levels of combined physical behaviors was the lowest in the Eastern Mediterranean. To date, few countries in the Eastern Mediterranean have commenced any comprehensive strategies for increasing the physical activity levels through sports, recreation, cycling, or walking in young adolescents [22]. In addition, the prevalence of engaging in active transportation was also the lowest in the Eastern Mediterranean. Because of cultural attitudes and beliefs, it is usually perceived that the pursuit of academic excellence has greater status than being physically active in this region [23]. The highest prevalence of high levels of combined physical behaviors was found in the Southeast Asia region, which included only four countries, largely contributed by the highest level of physical activity observed in Bangladesh (42.2%). Bangladesh is known as one of the poorest nations [24], and 80% of Bangladeshis live in rural areas. Here, adolescents are exceptionally active because most Bangladeshis earn a living through physical labor and many children help their parents with farm activities.

Another interesting finding of this study was the relationship between physical education and physical activity. School physical education is the only course that ensures all students participate in and increase physical activity. However, physical education is not included in the core curriculum in most countries [25]. Accumulating evidence has indicated that physical education is the most effective approach in maintaining a high level of physical activity in children and adolescents, as well as improving their motor skills, developing a positive disposition for an active lifestyle, and increasing direct engagement in moderate-to-vigorous physical activity [26-28]. In particular, Southeast Asia showed the lowest prevalence of physical education and the highest prevalence of physical activity. The Directorate of Technical Education Official states the reasons of low physical education in Asia as a lack of physical facilities, low motivation for physical education, and the lack of mandatory physical education [29]. In addition, the highest prevalence of physical education in the US may be because this region shows the highest prevalence of sedentary behavior (43.4%).

The physical activity level of girls differs significantly from that of boys, and the disparity is maintained into adulthood [30]. A meta-analysis of 26 prospective cohorts indicated a greater rate of decline in physical activity levels among girls than among boys [30]. In 2012, the data on children and adolescents in 105 countries reported by the Lancet physical activity research working group showed that the deficiency in physical activity increased with age, especially in girls [1]. Similar findings were observed in our study, wherein the pooled prevalence of physical activity was higher among boys than among girls (18.7%, 95% CI = 16.6%-20.9%, vs 11.7%, 95% CI = 10.8%-12.7%). Studies have found that biological factors, lack of enjoyment, and watching the latest variety shows increase the tendency for physical inactivity and sedentary behavior in girls [31,32]. In addition, girls are often overly protected and not encouraged to engage in physical activity by their families [33]. Moreover, compared with boys, girls have limited opportunities and facilities for physical activity [33]. Therefore, adolescent girls may need additional support and encouragement to maintain health-enhancing physical activity.

National or regional physical activity guidelines are regarded by WHO as the most basic steps to promote physical health [1], but at present, such guidelines for adolescents are absent or inadequate in most countries, especially in LMICs. Therefore, on the basis of WHO recommendations, different countries or regions should formulate physical activity guidelines in accordance with their social, cultural, and economic backgrounds to better promote adolescent health in their countries or regions [1]. Multi-component school-based interventions can increase physical activity among youth, such as providing enhanced PE; classroom activity breaks; and after-school activity space and equipment, encourage active transportation to school, and help adolescent building behavioral skills [34].

We used the most recent national GSHS data from 2009 to 2015, and the overall response rate was more than 98.56%. In addition, the use of a large sample size, standard procedures for the selection of participants, direct comparisons, and robust statistical methods to assess estimates of prevalence rates ensured that our findings were reliable and representative. Most previous studies have focused only on physical activity and have barely investigated sedentary behavior, physical education, and active transportation. To the best of our knowledge, this is the first study to estimate combined physical behaviors by considering the combined effect of these four behaviors in 12-15 year-old adolescents in LMICs. Our study provides a comprehensive picture of cross-country differences in physical behaviors in LMICs and can thus help countries formulate intervention programs to keep adolescents physically active.

### Limitations

Our study had the following limitations. First, GSHS is a self-reported survey mainly administered at schools and thus may be susceptible to recall bias. Second, the GSHS was not administered to older adolescents past school age, so the results may not be representative of this group of adolescents. Third, data on specific types of physical activity, frequency, intensity, type, and duration were not collected in the GSHS; thus, metabolic equivalents in adolescents could not be calculated. Fourthly, data on time spent on physical activity during physical education and active transportation were not available. Fifth, we observed high heterogeneity in the meta-analyses when pooling estimates across time and locations. However, heterogeneity can be overestimated when summarizing studies with large sample sizes. In addition, as we analyzed only 12-15 year-old adolescents, the possibility of variation across ages could not be ruled out. Lastly, although we used the latest released data, the data analyzed were at least five years old, and the surveys were conducted between a fairly long period of time (2009-2015).

## CONCLUSION

In conclusion, the prevalence of physical activity, active transportation, and physical education, as well as that of combined physical behaviors, was consistently low among young adolescents in LMICs. Integrated intervention strategies should be advocated to increase physical activity and thereby improve the physical health of adolescents in different countries and regions in accordance with their social, cultural, and economic backgrounds.

## Additional material

Online Supplementary Document
