# Supplementary material for: Physical behaviors of 12-15 year-old adolescents in 54 low- and middle-income countries: Results from the Global School-based Student Health Survey
Source: J Glob Health. 2020 May 2;10(1):010423. doi: 10.7189/jogh.10.010423 (PMC7211419; doi:10.7189/jogh.10.010423)
Supplement: Online Supplementary Document [file jogh-10-010423-s001.pdf]

Table S1. Combined physical behaviors among young adolescents aged 12-15 years by gender, region, and country groups.

	**Total, % (95% CI)**				**Boys, % (95% CI)**				**Girls, % (95% CI)**			
	Low	Moderate-low	Moderate-high	High	Low	Moderate-low	Moderate-high	High	Low	Moderate-low	Moderate-high	High
**Africa Region**												
Algeria*	17.5 (14.8-20.3)	41.5 (38.6-44.4)	34.5 (32.4-36.5)	6.5 (5.3-7.6)	15.6 (13.2-17.9)	40.1 (37.3-42.8)	34.9 (32.7-37.1)	9.4 (7.2-11.7)	19.5 (16.1-22.9)	42.6 (38.3-46.8)	33.9 (31.0-36.7)	4.1 (2.8-5.3)
Benin	5.5 (4.1-6.9)	28.4 (24.1-32.7)	43.2 (39.6-46.7)	22.9 (19.2-26.7)	4.9 (3.7-6.2)	27.2 (22.8-31.5)	44.2 (39.9-48.6)	23.7 (19.0-28.3)	7.0 (3.8-10.2)	30.8 (24.4-37.2)	41.3 (34.9-47.7)	20.9 (15.5-26.3)
Mauritania*	27.7 (23.1-32.3)	41.1 (37.2-45.0)	23.5 (19.7-27.3)	7.6 (5.3-10.0)	22.4 (17.9-26.8)	40.5 (36.1-45.0)	27.0 (21.0-33.0)	10.1 (6.6-13.6)	34.8 (27.1-42.5)	41.3 (36.1-46.4)	19.4 (14.7-24.1)	4.6 (2.6-6.5)
Mauritius*	20.1 (16.3-24.0)	46.7 (43.2-50.3)	27.4 (24.3-30.6)	5.7 (4.2-7.3)	15.0 (12.8-17.3)	45.6 (41.3-50.0)	31.7 (28.0-35.4)	7.7 (5.9-9.4)	24.8 (20.3-29.4)	47.8 (43.5-52.1)	23.5 (20.7-26.3)	3.9 (2.3-5.4)
Mozambique*	25.1 (16.9-33.3)	52.2 (48.0-56.4)	20.5 (15.3-25.7)	2.2 (0.5-3.8)	17.2 (10.8-23.7)	53.3 (45.6-61.0)	25.8 (20.4-31.2)	3.6 (1.5-5.8)	34.0 (23.8-44.3)	49.3 (45.4-53.3)	15.4 (7.2-23.6)	1.2 (0.0-2.8)
Namibia*	32.9 (29.0-36.8)	41.8 (39.0-44.7)	19.4 (17.1-21.6)	5.9 (4.4-7.4)	26.3 (22.1-30.5)	47.2 (43.8-50.6)	21.1 (18.6-23.9)	5.2 (3.1-7.4)	37.9 (33.0-42.8)	37.6 (33.2-42.0)	18.2 (15.0-21.3)	6.3 (4.7-7.9)
Seychelles	29.4 (26.6-32.2)	44.3 (41.7-46.8)	21.1 (18.9-23.3)	5.2 (4.1-6.3)	27.7 (23.8-31.5)	44.2 (40.9-47.6)	22.0 (18.7-25.3)	6.1 (4.2-8.0)	31.0 (27.8-34.1)	44.3 (41.0-47.5)	20.4 (18.2-22.7)	4.3 (3.0-5.7)
United Republic of Tanzania*	26.5 (22.8-30.2)	38.4 (36.0-40.9)	25.4 (22.3-28.5)	9.7 (8.3-11.1)	24.0 (19.2-28.8)	37.3 (33.2-41.4)	26.8 (22.5-31.0)	11.9 (9.5-14.4)	28.8 (24.2-33.4)	39.4 (36.1-42.6)	24.1 (20.8-27.3)	7.8 (6.1-9.4)
**Pooled estimates**	**23.0 (14.3-31.7)**	**41.8 (38.0-45.6)**	**26.9 (21.3-32.5)**	**7.8 (5.5-10.1)**	**19.0 (12.4-25.6)**	**41.7 (37.2-46.2)**	**29.2 (23.7-34.6)**	**9.3 (6.4-12.2)**	**27.0 (19.1-34.8)**	**41.9 (38.3-45.4)**	**24.5 (19.7-29.3)**	**5.7 (3.8-7.7)**
**I^2(%)**	98.4	91.2	96.7	94.5	97.6	90.3	94.6	91.5	96.1	83.0	93.4	91.0
**Region of the Americas**												

Antigua and Barbuda[*]	29.8 (25.6-34.0)	43.0 (39.5-46.5)	21.6 (17.8-25.4)	5.6 (3.6-7.6)	25.9 (21.4-30.4)	44.4 (38.5-50.3)	23.1 (17.8-28.4)	6.6 (4.1-9.2)	34.5 (28.9-40.1)	40.9 (36.9-44.9)	19.9 (16.6-23.1)	4.7 (2.4-7.0)
Argentina[*]	11.5 (10.3-12.7)	41.9 (40.1-43.7)	36.8 (35.0-38.5)	9.8 (8.7-10.9)	10.0 (8.6-11.4)	37.4 (35.1-39.7)	40.6 (38.5-42.8)	11.9 (10.2-13.6)	12.9 (11.4-14.4)	45.8 (43.9-47.7)	33.4 (31.3-35.6)	7.9 (6.8-9.0)
Barbados[*]	37.5 (33.9-41.0)	43.0 (39.8-46.2)	16.0 (13.8-18.2)	3.6 (2.4-4.7)	30.1 (25.2-35.0)	45.0 (41.0-48.9)	20.1 (16.5-23.6)	4.8 (3.1-6.6)	44.7 (39.8-49.5)	40.9 (35.9-45.8)	12.1 (9.6-14.5)	2.4 (1.3-3.5)
Belize[*]	24.9 (20.5-29.3)	37.7 (34.6-40.8)	29.9 (24.8-34.9)	7.5 (5.5-9.5)	21.0 (16.4-25.5)	37.8 (34.4-41.3)	31.1 (25.1-37.1)	10.1 (7.3-12.9)	28.5 (23.4-33.7)	37.6 (34.1-41.0)	28.8 (23.0-34.6)	5.1 (2.7-7.4)
Bolivia	16.5 (14.3-18.6)	41.4 (38.6-44.3)	33.0 (29.5-36.5)	9.1 (7.7-10.4)	17.6 (14.6-20.7)	39.6 (35.5-43.8)	33.6 (29.4-37.8)	9.1 (7.4-10.8)	15.8 (13.1-18.5)	43.5 (39.7-47.2)	31.6 (28.1-35.0)	9.2 (6.8-11.5)
Chile	15.7 (12.9-18.5)	42.4 (38.2-46.6)	32.5 (29.4-35.6)	9.4 (7.6-11.3)	13.9 (10.0-17.7)	40.9 (33.8-48.0)	33.4 (28.7-38.1)	11.8 (8.5-15.1)	17.4 (13.5-21.4)	43.4 (38.8-48.0)	31.8 (27.5-36.1)	7.4 (5.2-9.6)
Bahamas[*]	35.2 (31.4-39.1)	45.4 (42.3-48.5)	16.5 (13.5-19.5)	2.9 (1.9-3.8)	30.3 (24.5-36.1)	47.8 (42.4-53.3)	17.4 (13.6-21.3)	4.5 (2.5-6.4)	39.4 (34.6-44.1)	43.3 (38.0-48.6)	15.7 (12.2-19.1)	1.7 (1.1-2.3)
Costa Rica[*]	19.5 (16.0-23.0)	41.5 (38.7-44.4)	29.8 (27.2-32.5)	9.1 (7.8-10.5)	15.7 (12.0-19.4)	39.7 (35.7-43.7)	32.2 (29.2-35.2)	12.4 (10.1-14.7)	23.4 (19.1-27.6)	43.2 (39.5-47.0)	27.5 (23.0-32.0)	5.9 (4.7-7.1)
Curaçao[*]	33.8 (30.4-37.2)	44.4 (42.0-46.7)	17.4 (13.8-21.0)	4.4 (3.3-5.6)	30.6 (26.2-34.9)	42.3 (39.1-45.4)	20.7 (16.1-25.4)	6.4 (4.7-8.2)	36.7 (32.6-40.9)	46.6 (43.2-50.1)	14.1 (11.0-17.3)	2.5 (1.4-3.5)
El Salvador[*]	19.4 (15.5-23.3)	42.0 (39.3-44.7)	31.0 (26.8-35.3)	7.6 (5.5-9.7)	17.9 (12.7-23.0)	40.0 (35.2-44.8)	32.8 (27.0-38.5)	9.4 (6.4-12.3)	21.2 (16.6-25.7)	44.2 (40.8-47.5)	28.9 (24.2-33.7)	5.7 (4.0-7.5)
Guatemala	19.6 (16.1-23.0)	43.7 (40.0-47.3)	31.1 (27.5-34.8)	5.6 (3.6-7.7)	18.5 (15.6-21.4)	44.1 (39.1-49.1)	32.2 (27.7-36.6)	5.2 (2.2-8.2)	21.1 (16.1-26.1)	43.8 (40.2-47.4)	29.2 (24.3-34.1)	5.9 (4.2-7.6)
Guyana[*]	34.9 (29.7-40.1)	45.8 (43.2-48.4)	16.2 (12.1-20.4)	3.1 (2.0-4.2)	30.2 (25.0-35.4)	47.6 (43.3-51.9)	17.8 (13.5-22.0)	4.4 (3.0-5.9)	38.8 (32.0-45.7)	44.7 (40.7-48.7)	14.7 (9.3-20.1)	1.8 (0.9-2.7)
Honduras[*]	16.4 (12.9-19.8)	43.9 (39.9-47.8)	32.2 (27.5-36.8)	7.6 (6.0-9.2)	13.6 (10.5-16.8)	42.2 (36.5-48.0)	35.0 (29.0-41.0)	9.2 (6.5-11.8)	18.8 (14.2-23.4)	45.5 (40.7-50.4)	29.5 (23.1-35.8)	6.2 (4.4-8.0)
Peru	18.0	43.6	32.9	5.5	18.6	44.2	31.9	5.3	17.6	43.2	33.7	5.5

	(15.0-21.1)	(41.2-46.1)	(29.4-36.3)	(4.3-6.6)	(15.2-22.0)	(41.1-47.4)	(28.2-35.6)	(3.9-6.7)	(13.7-21.5)	(39.8-46.6)	(29.1-38.3)	(3.9-7.1)
Suriname	24.0	41.6	27.5	6.9	21.3	42.7	27.5	8.6	26.2	40.5	27.7	5.6
	(18.3-29.7)	(38.5-44.8)	(22.7-32.3)	(5.1-8.7)	(16.0-26.5)	(38.5-46.8)	(24.3-30.6)	(5.7-11.5)	(19.5-32.9)	(35.4-45.6)	(21.1-34.4)	(3.7-7.5)
Trinidad and	29.6	45.7	20.3	4.4	25.4	46.5	22.0	6.1	33.9	44.8	18.6	2.8
Tobago*	(25.9-33.3)	(42.5-48.8)	(18.4-22.3)	(3.3-5.5)	(20.8-30.0)	(43.0-50.1)	(19.1-24.8)	(4.2-8.0)	(29.2-38.6)	(39.0-50.5)	(16.7-20.5)	(1.6-3.9)
Uruguay*	11.7	40.3	37.1	10.9	8.4	34.9	41.5	15.2	14.6	44.9	33.1	7.3
	(9.7-13.7)	(37.3-43.4)	(33.9-40.2)	(9.0-12.8)	(6.2-10.6)	(30.6-39.1)	(37.8-45.2)	(12.0-18.4)	(11.7-17.6)	(42.0-47.8)	(29.5-36.7)	(6.2-8.5)
**Pooled**	**23.3**	**42.8**	**27.1**	**6.6**	**20.3**	**42.1**	**29.0**	**8.2**	**26.0**	**44.5**	**25.2**	**5.1**
**estimates**	**(19.1-27.5)**	**(41.8-43.8)**	**(23.1-31.2)**	**(5.3-7.9)**	**(16.8-23.8)**	**(40.2-43.9)**	**(25.0-33.0)**	**(6.7-9.6)**	**(2.2-30.9)**	**(42.4-44.7)**	**(21.1-29.3)**	**(3.9-6.3)**
**I^2(%)**	97.1	47.4	96.5	93.2	94.6	71.8	94.7	88.0	96.4	38.1	95.6	93.3
**Eastern Mediterranean Region**												
Afghanistan	14.5	42.8	34.8	7.9	13.2	40.0	38.1	8.7	15.6	48.3	29.3	6.8
	(11.5-17.4)	(36.2-49.3)	(26.8-42.9)	(5.7-10.2)	(8.7-17.7)	(31.3-48.8)	(26.2-49.9)	(5.0-12.4)	(11.5-19.7)	(40.8-55.9)	(22.6-35.9)	(4.7-9.0)
Egypt	23.1	47.2	25.8	4.0	18.7	47.2	29.5	4.7	27.0	47.5	22.3	3.2
	(18.8-27.4)	(41.6-52.7)	(19.7-31.9)	(2.3-5.6)	(13.7-23.6)	(40.5-54.0)	(21.4-37.5)	(2.8-6.6)	(20.3-33.8)	(40.2-54.8)	(14.6-29.9)	(1.6-4.8)
Iraq*	26.3	39.1	26.4	8.3	19.4	37.4	31.5	11.7	34.6	41.1	20.1	4.1
	(22.4-30.1)	(35.8-42.3)	(22.7-30.1)	(6.0-10.5)	(17.0-21.7)	(33.2-41.6)	(27.0-36.1)	(8.6-14.8)	(28.4-40.9)	(37.6-44.7)	(16.3-23.9)	(1.7-6.5)
Kuwait*	33.2	50.1	14.0	2.7	30.3	51.2	14.8	3.6	36.2	48.7	13.2	1.9
	(28.7-37.7)	(45.8-54.3)	(10.7-17.3)	(1.3-4.2)	(22.9-37.6)	(43.6-58.9)	(12.1-17.6)	(1.5-5.7)	(34.0-38.4)	(46.1-51.4)	(9.6-16.8)	(1.0-2.7)
Lebanon*	27.8	47.1	21.9	3.2	23.0	47.3	26.1	3.7	32.0	46.9	18.1	2.9
	(25.3-30.2)	(44.0-50.2)	(17.6-26.1)	(2.2-4.3)	(20.2-25.7)	(40.4-54.2)	(20.2-32.0)	(2.4-4.9)	(27.5-36.5)	(44.5-49.3)	(14.5-21.8)	(1.7-4.1)
Morocco*	14.6	37.1	38.3	10.1	13.7	37.0	37.3	12.1	16.2	37.2	38.8	7.8
	(12.3-16.9)	(33.8-40.3)	(33.6-42.9)	(8.4-11.8)	(10.9-16.4)	(34.2-39.9)	(32.8-41.7)	(9.5-14.6)	(12.8-19.5)	(32.0-42.4)	(32.4-45.2)	(6.2-9.4)
Oman*	24.9	52.0	19.8	3.3	20.5	50.9	23.8	4.8	28.6	53.4	16.2	1.9
	(21.5-28.2)	(49.4-54.7)	(16.9-22.7)	(2.5-4.1)	(18.0-23.0)	(48.3-53.5)	(21.0-26.5)	(3.7-5.9)	(24.6-32.7)	(50.0-56.7)	(13.3-19.0)	(1.0-2.7)

Pakistan*	28.0 (21.2-34.7)	42.0 (38.9-45.1)	23.0 (18.7-27.4)	7.0 (3.2-10.8)	18.3 (14.7-21.9)	43.4 (38.6-48.2)	28.6 (25.0-32.3)	9.7 (4.3-15.0)	42.8 (36.8-48.8)	39.8 (37.1-42.6)	14.5 (9.9-19.1)	2.9 (2.1-3.6)
Qatar	29.9 (26.7-33.1)	43.9 (39.7-48.1)	20.9 (17.6-24.2)	5.3 (3.4-7.2)	26.3 (21.9-30.6)	44.7 (38.1-51.2)	22.6 (17.0-28.1)	6.5 (4.7-8.4)	33.1 (28.5-37.7)	43.2 (38.1-48.2)	19.5 (15.7-23.4)	4.2 (1.2-7.2)
Sudan*	38.8 (32.9-44.8)	45.7 (41.2-50.2)	13.7 (10.0-17.4)	1.8 (0.9-2.7)	32.5 (28.3-36.7)	49.0 (42.6-55.5)	16.1 (11.5-20.7)	2.4 (0.7-4.1)	45.9 (37.9-53.9)	42.0 (36.8-47.1)	10.9 (7.1-14.8)	1.2 (0.5-1.9)
Syrian Arab Republic	27.7 (23.6-31.8)	43.3 (39.8-46.9)	23.7 (20.5-26.8)	5.3 (3.9-6.7)	25.9 (22.8-29.1)	42.1 (40.4-43.8)	25.5 (22.6-28.5)	6.4 (5.2-7.7)	29.6 (23.5-35.7)	44.6 (38.6-50.6)	21.7 (18.2-25.3)	4.1 (2.4-5.8)
United Arab Emirates*	33.8 (29.3-38.3)	49.3 (45.7-52.9)	15.0 (12.5-17.5)	1.9 (1.0-2.7)	25.6 (22.2-29.0)	50.3 (46.6-53.9)	21.2 (18.4-24.0)	2.9 (1.9-3.9)	39.0 (35.2-42.9)	48.7 (44.9-52.5)	10.9 (9.1-12.7)	1.3 (0.6-2.0)
**Pooled estimates**	**26.7 (22.4-31.0)**	**45.0 (42.1-47.9)**	**22.7 (19.0-26.5)**	**4.9 (3.5-6.2)**	**22.1 (19.1-25.1)**	**44.9 (41.6-48.2)**	**25.7 (21.8-29.5)**	**6.0 (4.5-7.5)**	**31.5 (26.2-36.9)**	**45.1 (42.3-47.9)**	**19.1 (15.5-22.8)**	**3.3 (2.3-4.3)**
**I^2(%)**	94.2	86.4	91.8	91.7	89.3	87.0	90.7	88.2	94.5	83.5	91.3	88.3
**Southeast Asia Region**												
Bangladesh	10.4 (7.4-13.3)	21.9 (19.0-24.9)	34.1 (30.1-38.1)	33.6 (27.2-40.0)	10.5 (5.5-15.4)	19.1 (15.0-23.3)	36.3 (31.4-41.1)	34.1 (25.4-42.8)	10.4 (7.0-13.8)	26.7 (22.8-30.6)	30.0 (25.8-34.3)	32.9 (25.7-40.1)
Indonesia*	32.3 (28.9-35.7)	41.6 (39.4-43.8)	21.4 (18.0-24.7)	4.7 (3.5-6.0)	30.5 (27.0-33.9)	42.0 (39.5-44.5)	21.8 (18.7-25.0)	5.7 (4.1-7.3)	34.1 (30.4-37.9)	41.3 (38.7-43.8)	20.8 (17.0-24.6)	3.8 (2.6-5.0)
Thailand	32.3 (29.6-35.1)	47.3 (45.3-49.2)	16.7 (14.8-18.6)	3.7 (2.6-4.8)	31.7 (27.7-35.7)	46.0 (43.4-48.6)	17.3 (14.6-19.9)	5.0 (3.1-6.9)	33.0 (28.9-37.1)	48.4 (45.0-51.9)	16.1 (13.5-18.7)	2.4 (1.6-3.3)
Timor-Leste*	30.4 (27.3-33.5)	51.3 (49.4-53.1)	14.7 (12.5-16.8)	3.6 (2.7-4.6)	27.7 (22.8-32.6)	51.4 (48.4-54.5)	15.9 (12.9-18.9)	5.0 (3.4-6.6)	33.1 (29.9-36.3)	49.9 (47.4-52.4)	14.1 (11.7-16.5)	2.9 (1.8-4.0)
**Pooled estimates**	**26.3 (15.7-37.0)**	**40.6 (29.9-51.3)**	**21.5 (14.9-28.2)**	**8.6 (4.9-12.3)**	**25.2 (16.3-34.1)**	**39.7 (28.8-50.6)**	**22.6 (15.5-29.6)**	**8.9 (4.8-13.0)**	**27.6 (15.9-39.3)**	**41.6 (32.7-50.6)**	**20.0 (14.0-26.1)**	**6.7 (3.4-10.0)**
**I^2(%)**	98.0	99.0	96.1	96.4	94.2	87.0	94.6	92.9	97.6	97.2	93.3	95.7
**Western**												

**Pacific**												
**Region**												
Brunei	34.4	49.4	14.4	1.8	29.4	49.2	18.6	2.8	39.0	49.5	10.7	0.8
Darussalam*	(31.9-37.0)	(46.7-52.2)	(12.4-16.3)	(1.1-2.4)	(26.2-32.5)	(45.3-53.2)	(15.8-21.3)	(1.5-4.1)	(35.2-42.7)	(46.1-53.0)	(8.7-12.7)	(0.2-1.3)
Cambodia*	19.6	43.4	31.0	6.0	19.7	40.8	31.5	7.9	19.7	45.6	30.4	4.3
	(16.3-23.0)	(40.8-45.9)	(27.1-34.8)	(4.8-7.3)	(15.4-23.9)	(36.6-45.1)	(27.3-35.8)	(6.2-9.6)	(15.9-23.6)	(42.6-48.5)	(25.4-35.4)	(2.3-6.2)
Kiribati	20.5	43.1	25.4	10.9	18.4	41.6	26.8	13.2	22.5	44.1	24.3	9.0
	(18.3-22.8)	(39.2-47.1)	(22.3-28.6)	(8.4-13.5)	(15.2-21.5)	(37.4-45.9)	(21.9-31.7)	(9.5-16.9)	(19.3-25.8)	(38.0-50.2)	(20.8-27.8)	(6.1-12.0)
Lao People's	25.6	48.1	21.5	4.7	21.1	48.3	24.0	6.7	29.9	48.2	19.1	2.9
Democratic	(21.6-29.6)	(45.4-50.8)	(17.4-25.6)	(3.2-6.2)	(17.7-24.4)	(44.1-52.4)	(20.2-27.8)	(4.3-9.1)	(24.1-35.6)	(43.9-52.5)	(13.9-24.2)	(1.7-4.1)
Republic*												
Malaysia*	22.2	45.6	25.3	7.0	21.0	42.1	27.7	9.3	23.3	49.1	22.9	4.7
	(20.5-23.9)	(44.1-47.1)	(23.5-27.1)	(6.0-7.9)	(18.9-23.1)	(40.3-43.9)	(25.7-29.7)	(8.0-10.6)	(21.5-25.1)	(47.1-51.1)	(20.6-25.2)	(3.9-5.5)
Mongolia	8.2	32.0	44.8	15.0	8.7	31.3	43.5	16.6	7.7	32.8	45.9	13.6
	(6.7-9.6)	(29.2-34.9)	(42.2-47.3)	(13.1-17.0)	(7.0-10.4)	(28.1-34.5)	(40.2-46.7)	(14.2-18.9)	(5.9-9.5)	(29.1-36.5)	(42.4-49.4)	(11.2-16.0)
Philippines	24.2	48.0	23.6	4.2	26.5	46.1	23.2	4.2	22.2	49.7	24.0	4.1
	(21.6-26.8)	(45.5-50.5)	(21.1-26.1)	(3.2-5.1)	(23.8-29.1)	(42.6-49.6)	(20.1-26.3)	(3.2-5.3)	(18.7-25.7)	(47.3-52.1)	(21.4-26.5)	(2.9-5.3)
Samoa	28.1	46.3	20.0	5.5	26.9	49.4	18.8	4.8	28.9	43.9	21.2	5.9
	(25.4-30.9)	(43.6-49.1)	(18.0-22.0)	(4.2-6.8)	(23.6-30.3)	(45.5-53.3)	(15.4-22.3)	(2.5-7.2)	(25.6-32.3)	(40.0-47.9)	(19.0-23.4)	(3.9-8.0)
Solomon	20.0	44.6	27.9	7.5	21.1	42.0	28.6	8.2	17.6	46.0	30.1	6.3
Islands	(14.1-26.0)	(38.1-51.2)	(22.0-33.8)	(4.1-10.9)	(13.8-28.5)	(34.8-49.3)	(22.7-34.6)	(3.4-12.9)	(11.1-24.0)	(40.4-51.6)	(22.3-37.9)	(4.3-8.4)
Tonga	28.7	43.7	20.2	7.3	27.6	43.6	22.2	6.7	30.1	43.8	18.2	7.9
	(26.1-31.4)	(41.1-46.3)	(17.8-22.6)	(5.9-8.8)	(24.1-31.1)	(40.1-47.0)	(18.1-26.3)	(4.8-8.5)	(26.2-33.9)	(40.1-47.4)	(15.6-20.9)	(6.1-9.8)
Vanuatu	13.4	31.6	46.4	8.6	11.4	32.5	47.3	8.8	15.5	30.9	45.5	8.1
	(4.6-22.2)	(23.9-39.3)	(33.5-59.4)	(3.7-13.4)	(3.2-19.6)	(24.5-40.6)	(37.1-57.6)	(3.1-14.4)	(5.8-25.2)	(21.2-40.6)	(28.0-63.0)	(2.5-13.7)
Vietnam*	6.5	33.7	49.1	10.7	6.1	32.3	49.3	12.2	6.8	34.9	48.9	9.4
	(4.9-8.1)	(29.9-37.5)	(44.4-53.8)	(8.9-12.5)	(4.2-8.0)	(27.1-37.5)	(44.2-54.5)	(9.3-15.2)	(4.4-9.3)	(30.6-39.1)	(43.4-54.5)	(6.9-11.9)

Wallis and Futuna	22.3 (18.5-26.0)	51.7 (47.0-56.5)	20.6 (17.7-23.5)	5.4 (3.6-7.1)	21.1 (15.8-26.4)	50.8 (44.5-57.0)	21.6 (17.8-25.4)	6.6 (3.7-9.4)	23.8 (18.6-29.1)	52.1 (45.7-58.6)	19.8 (15.6-24.1)	4.2 (1.8-6.6)
**Pooled estimates**	**21.1 (15.8-26.5)**	**43.4 (40.5-46.4)**	**27.9 (22.8-33.1)**	**7.2 (5.3-9.0)**	**20.0 (15.0-24.9)**	**42.5 (39.2-45.8)**	**29.1 (24.2-33.9)**	**8.2 (6.1-10.3)**	**22.1 (16.5-27.7)**	**44.3 (41.0-47.5)**	**27.0 (21.5-32.6)**	**6.1 (4.2-7.9)**
**I^2(%)**	98.3	92.3	97.7	95.9	97.2	89.1	95.6	93.0	97.4	89.5	97.2	95.1
**Total**												
**Pooled estimates[*]**	**24.1 (21.6-26.5)**	**43.0 (42.1-43.9)**	**25.3 (22.8-27.7)**	**6.6 (5.4-7.8)**	**21.1 (19.2-23.0)**	**42.6 (41.2-43.9)**	**27.5 (25.4-29.6)**	**7.8 (6.5-9.0)**	**26.9 (22.8-31.1)**	**44.3 (43.4-45.3)**	**23.1 (19.9-26.2)**	**5.1 (3.8-6.4)**
**I^2(%)**	0	0	22.3	52.0	0	0	0.7	43.2	29.7	0	54.4	67.9

[*]There was significantly difference in the prevalence of joint physical behavior between different sex group (P<0.05).

Table S2. Combined physical behaviors among young adolescents aged 12-15 years by age, region, and country groups.

	**12-13 years, % (95% CI)**				**14-15 years, % (95% CI)**			
	Low	Moderate-low	Moderate-high	High	Low	Moderate-low	Moderate-high	High
**Africa Region**								
Algeria*	17.9 (14.2-21.6)	43.9 (39.4-48.4)	31.7 (28.5-34.9)	6.4 (5.1-7.8)	17.2 (14.4-20.0)	39.4 (37.1-41.7)	36.9 (34.9-38.9)	6.5 (4.9-8.1)
Benin	6.7 (2.8-10.5)	26.9 (20.8-33.1)	45.3 (38.6-51.9)	21.2 (16.9-25.4)	5.2 (3.5-6.9)	28.8 (23.6-33.9)	42.6 (39.0-46.2)	23.4 (19.5-27.4)
Mauritania*	25.9 (22.9-28.9)	44.1 (38.7-49.6)	23.1 (16.2-30.1)	6.8 (3.2-10.4)	28.3 (22.9-33.8)	40.1 (35.5-44.8)	23.6 (19.5-27.8)	7.9 (5.4-10.3)
Mauritius*	21.2 (16.2-26.2)	47.6 (42.0-53.2)	26.3 (24.2-28.5)	4.9 (2.6-7.3)	19.4 (14.4-24.4)	46.2 (41.9-50.5)	28.1 (23.5-32.8)	6.2 (4.6-7.9)
Mozambique*	23.1 (16.1-30.2)	50.2 (39.1-61.4)	25.7 (15.8-35.5)	1.0 (0.0-2.2)	25.9 (16.4-35.5)	53.0 (48.3-57.8)	18.3 (12.0-24.6)	2.7 (0.5-4.9)
Namibia*	33.7 (27.5-39.9)	41.7 (37.8-45.6)	18.5 (13.9-23.1)	6.1 (4.6-7.7)	32.6 (28.6-36.6)	41.9 (38.8-45.0)	19.7 (17.4-22.1)	5.8 (4.1-7.4).
Seychelles	28.4 (23.9-32.9)	47.1 (43.3-50.8)	19.5 (16.4-22.6)	5.0 (3.4-6.7)	30.4 (27.1-33.8)	41.5 (38.6-44.5)	22.7 (19.6-25.8)	5.3 (3.8-6.8)
United Republic of Tanzania*	27.8 (23.8-31.9)	42.4 (39.6-45.1)	20.9 (18.3-23.6)	8.9 (7.1-10.7)	25.4 (20.5-30.2)	35.0 (32.0-38.0)	29.3 (25.2-33.4)	10.3 (8.3-12.4)
**Pooled estimates**	**23.0 (17.1-28.8)**	**42.8 (39.0-46.5)**	**26.1 (21.5-30.7)**	**7.2 (4.4-9.9)**	**22.9 (14.2-31.7)**	**40.7 (36.9-44.6)**	**27.7 (21.4-34.1)**	**8.1 (5.6-10.7)**
**I^2(%)**	93.1	80.6	91.6	94.7	97.9	89.7	96.6	92.9

**Region of the Americas**								
Antigua and Barbuda*	29.6 (23.6-35.7)	43.7 (38.3-49.1)	21.0 (17.0-25.0)	5.7 (3.5-7.9)	29.9 (25.2-34.7)	42.6 (38.3-46.9)	22.0 (16.9-27.0)	5.5 (3.3-7.8)
Argentina*	12.3 (10.2-14.4)	40.8 (37.7-43.9)	36.4 (33.3-39.5)	10.5 (8.3-12.6)	11.2 (9.9-12.4)	42.4 (40.9-44.0)	36.9 (35.4-38.5)	9.5 (8.5-10.5)
Barbados*	35.8 (29.0-42.5)	42.5 (34.8-50.3)	17.0 (11.7-22.3)	4.7 (2.5-6.9)	38.0 (34.0-42.0)	43.1 (39.6-46.6)	15.7 (13.2-18.2)	3.2 (1.9-4.4)
Belize*	26.3 (20.7-32.0)	35.8 (32.1-39.5)	31.0 (24.5-37.5)	6.9 (4.8-9.0)	23.6 (19.4-27.9)	39.5 (35.7-43.2)	28.8 (24.2-33.5)	8.1 (5.3-10.9)
Bolivia	18.4 (15.8-21.0)	43.2 (40.0-46.4)	28.5 (25.2-31.7)	9.9 (7.6-12.3)	15.6 (12.8-18.4)	40.6 (36.7-44.6)	35.1 (30.3-39.9)	8.7 (7.1-10.3)
Chile	16.9 (13.1-20.7)	39.7 (35.6-43.7)	34.0 (30.4-37.5)	9.5 (6.9-12.0)	14.9 (11.6-18.3)	44.1 (37.9-50.4)	31.6 (27.3-35.9)	9.4 (7.4-11.4)
Bahamas*	34.9 (30.3-39.5)	47.1 (43.3-50.9)	15.0 (12.1-17.8)	3.0 (1.8-4.3)	35.6 (29.4-41.8)	43.5 (38.9-48.1)	18.2 (12.9-23.5)	2.7 (1.5-3.9)
Costa Rica*	21.2 (16.7-25.8)	43.8 (39.7-48.0)	25.8 (23.3-28.2)	9.2 (6.8-11.5)	18.7 (14.9-22.5)	40.4 (37.0-43.8)	31.9 (29.0-34.8)	9.1 (7.0-11.1)
Curaçao*	33.2 (28.2-38.1)	47.0 (42.2-51.8)	16.1 (11.2-21.0)	3.7 (2.0-5.5)	34.1 (30.4-37.8)	43.2 (40.9-45.5)	17.9 (14.2-21.7)	4.7 (3.2-6.2)
El Salvador*	21.5 (15.3-27.6)	36.8 (30.8-42.8)	36.4 (30.0-42.8)	5.3 (1.7-8.9)	18.5 (14.4-22.5)	44.4 (41.5-47.4)	28.5 (24.1-32.9)	8.6 (6.5-10.8)
Guatemala	22.4 (15.4-29.3)	45.5 (39.3-51.7)	28.1 (24.3-31.9)	4.1 (2.2-6.0)	18.0 (14.1-22.0)	42.6 (37.5-47.8)	32.8 (28.2-37.5)	6.5 (4.1-8.9)
Guyana*	30.1 (23.4-36.8)	50.8 (47.8-53.9)	16.2 (11.3-21.1)	2.9 (1.5-4.3)	36.8 (31.9-41.8)	43.7 (41.0-46.4)	16.3 (11.7-20.8)	3.2 (1.9-4.4)
Honduras*	17.0	44.0	30.5	8.5	15.7	43.8	33.7	6.8

	(11.2-22.8)	(37.5-50.4)	(23.6-37.4)	(5.5-11.5)	(12.0-19.5)	(40.6-47.0)	(29.0-38.3)	(4.9-8.7)
Peru	18.6	44.4	30.7	6.3	17.9	43.4	33.6	5.2
	(14.1-23.1)	(38.6-50.3)	(23.3-38.1)	(4.4-8.1)	(14.5-21.2)	(41.1-45.6)	(30.3-36.9)	(3.9-6.4)
Suriname	26.3	41.0	24.0	8.7	22.9	41.9	29.2	6.0
	(19.9-32.7)	(35.2-46.8)	(19.8-28.1)	(5.2-12.2)	(16.4-29.3)	(38.3-45.5)	(22.6-35.8)	(4.3-7.7)
Trinidad and	29.3	47.2	19.2	4.2	29.9	44.3	21.2	4.6
Tobago*	(24.6-33.9)	(43.9-50.6)	(16.8-21.7)	(2.4-6.0)	(26.5-33.2)	(40.3-48.4)	(18.4-24.0)	(3.0-6.2)
Uruguay*	9.6	38.5	40.0	12.0	12.5	41.1	36.0	10.4
	(7.0-12.3)	(34.0-42.9)	(35.2-44.8)	(9.5-14.4)	(10.3-14.8)	(37.8-44.3)	(33.1-38.8)	(8.2-12.7)
**Pooled estimates**	**23.5**	**43.1**	**26.3**	**6.7**	**23.0**	**42.7**	**27.6**	**6.5**
	**(19.5-27.6)**	**(41.0-45.3)**	**(22.6-30.1)**	**(5.2-8.1)**	**(18.7-27.3)**	**(41.9-43.4)**	**(23.6-31.7)**	**(5.2-7.8)**
**I^2(%)**	93.5	75.8	93.6	87.3	96.7	0.0	95.7	90.9
**Eastern Mediterranean Region**								
Afghanistan	18.1	44.6	28.4	8.8	13.0	42.1	37.3	7.6
	(13.1-23.1)	(34.7-54.6)	(20.1-36.7)	(4.1-13.6)	(9.8-16.3)	(35.9-48.2)	(28.6-46.0)	(5.1-10.1)
Egypt	20.8	49.5	25.6	4.1	25.6	44.5	26.0	3.8
	(14.4-27.2)	(42.5-56.4)	(18.4-32.7)	(1.7-6.5)	(19.3-31.9)	(37.7-51.3)	(17.7-34.4)	(2.2-5.4)
Iraq*	23.4	42.2	26.5	7.9	28.0	37.2	26.4	8.5
	(18.9-27.8)	(37.5-46.9)	(21.9-31.1)	(4.8-11.0)	(23.1-32.8)	(33.8-40.6)	(22.1-30.6)	(6.1-10.9)
Kuwait*	30.1	52.2	13.8	3.9	34.5	49.1	14.1	2.2
	(24.4-35.8)	(45.6-58.8)	(9.2-18.4)	(2.6-5.2)	(28.9-40.2)	(44.8-53.4)	(10.8-17.4)	(0.9-3.5)
Lebanon*	27.9	48.6	20.3	3.2	27.7	46.0	23.0	3.3
	(25.1-30.8)	(45.9-51.4)	(16.7-23.8)	(2.0-4.3)	(23.7-31.6)	(41.6-50.5)	(16.7-29.2)	(1.8-4.9)
Morocco*	15.8	34.7	40.3	9.1	13.6	38.9	36.7	10.8
	(12.6-19.0)	(30.8-38.7)	(34.5-46.1)	(6.6-11.6)	(10.9-16.4)	(35.2-42.6)	(31.3-42.0)	(8.6-13.1)

Oman*	24.6 (20.3-28.8)	50.9 (46.1-55.7)	21.2 (17.3-25.1)	3.3 (1.9-4.7)	25.0 (21.1-28.9)	52.4 (49.7-55.0)	19.4 (15.9-22.8)	3.3 (2.3-4.2)
Pakistan*	32.5 (24.1-40.9)	43.7 (38.6-48.7)	17.5 (12.3-22.6)	6.4 (2.6-10.1)	26.6 (20.0-33.1)	41.5 (38.1-44.8)	24.8 (20.5-29.0)	7.2 (3.3-11.2)
Qatar	29.5 (25.0-34.1)	43.3 (38.7-48.0)	20.4 (17.5-23.3)	6.7 (3.5-9.8)	30.3 (25.8-34.8)	44.6 (38.2-51.0)	21.4 (16.3-26.5)	3.7 (2.1-5.3)
Sudan*	39.2 (31.7-46.7)	46.2 (39.7-52.7)	12.4 (7.9-17.0)	2.1 (0.3-4.0)	38.7 (32.0-45.4)	45.5 (40.4-50.6)	14.1 (9.2-19.0)	1.7 (0.8-2.6)
Syrian Arab Republic	29.1 (23.9-34.3)	44.8 (39.6-50.0)	21.9 (18.9-24.8)	4.3 (3.3-5.3)	26.5 (22.4-30.7)	42.1 (38.8-45.3)	25.3 (21.1-29.4)	6.2 (4.0-8.3)
United Arab Emirates*	28.9 (25.3-32.4)	52.5 (48.8-56.3)	17.3 (14.7-20.0)	1.3 (0.5-2.0)	36.1 (30.5-41.7)	47.8 (43.6-52.1)	13.9 (10.8-17.0)	2.1 (1.1-3.2)
**Pooled estimates**	**26.3 (22.8-29.8)**	**46.0 (42.8-49.3)**	**21.7 (18.4-25.0)**	**4.6 (3.3-5.9)**	**26.9 (22.0-31.9)**	**44.3 (41.2-47.4)**	**23.1 (19.0-27.2)**	**4.8 (3.4-6.1)**
**I^2(%)**	85.5	81.6	87.1	85.4	93.3	85.1	89.7	89.3
**Southeast Asia Region**								
Bangladesh	18.5 (13.3-23.7)	26.2 (20.0-32.4)	29.9 (25.9-34.0)	25.4 (18.8-32.0)	6.8 (4.5-9.2)	20.1 (17.5-22.7)	35.9 (30.6-41.2)	37.2 (29.6-44.8)
Indonesia*	33.3 (29.6-37.0)	40.7 (38.3-43.1)	20.9 (17.5-24.2)	5.1 (3.7-6.5)	31.2 (28.2-34.3)	42.5 (39.9-45.2)	21.9 (18.8-25.0)	4.4 (2.9-5.9)
Thailand	32.2 (28.7-35.8)	46.6 (43.1-50.2)	16.9 (14.4-19.5)	4.2 (2.9-5.6)	32.4 (28.9-35.8)	47.8 (44.7-50.9)	16.5 (14.2-18.8)	3.3 (1.9-4.7)
Timor-Leste*	30.2 (25.4-35.0)	53.5 (48.7-58.2)	12.4 (9.0-15.8)	4.0 (2.1-5.8)	30.5 (27.2-33.8)	50.4 (48.0-52.7)	15.6 (13.6-17.6)	3.5 (2.6-4.5)
**Pooled estimates**	**28.8 (22.9-34.7)**	**42.0 (33.7-50.2)**	**19.9 (13.5-26.4)**	**7.5 (4.0-10.9)**	**25.2 (11.4-39.0)**	**40.2 (26.3-54.1)**	**22.0 (15.9-28.1)**	**8.6 (4.5-12.7)**

**I^2(%)**	87.1	94.5	93.5	92.3	98.8	99.1	94.7	96.0
**Western Pacific Region**								
Brunei Darussalam*	30.4 (26.9-34.0)	54.5 (50.6-58.4)	12.1 (9.6-14.7)	3.0 (1.5-4.4)	36.3 (33.3-39.2)	47.1 (44.0-50.3)	15.4 (13.0-17.8)	1.2 (0.6-1.8)
Cambodia*	20.9 (17.0-24.8)	44.4 (40.7-48.2)	28.3 (21.7-34.9)	6.3 (3.6-9.1)	19.1 (15.5-22.8)	42.9 (39.9-46.0)	32.0 (28.5-35.5)	5.9 (4.5-7.3)
Kiribati	23.1 (19.1-27.0)	43.1 (37.1-49.1)	24.6 (20.4-28.8)	9.2 (5.8-12.6)	19.4 (17.1-21.8)	43.1 (38.7-47.5)	25.7 (22.2-29.2)	11.7 (8.7-14.7)
Lao People's Democratic Republic*	20.1 (13.6-26.5)	46.7 (39.5-53.8)	28.8 (22.3-35.3)	4.4 (0.9-8.0)	26.4 (21.6-31.2)	48.3 (45.2-51.4)	20.5 (16.1-24.9)	4.8 (3.2-6.3)
Malaysia*	20.8 (18.9-22.7)	46.5 (44.7-48.3)	25.5 (23.4-27.7)	7.2 (5.8-8.6)	22.9 (20.8-25.0)	45.2 (43.3-47.0)	25.1 (23.0-27.2)	6.8 (5.8-7.8)
Mongolia	7.3 (5.6-9.1)	31.2 (27.5-35.0)	44.9 (41.3-48.4)	16.6 (13.9-19.2)	8.8 (7.1-10.5)	32.7 (29.4-36.0)	44.7 (42.1-47.2)	13.8 (11.2-16.4)
Philippines	24.5 (21.1-28.0)	47.9 (45.4-50.5)	23.9 (20.3-27.6)	3.6 (2.6-4.6)	24.0 (21.3-26.8)	48.0 (44.6-51.5)	23.4 (20.7-26.1)	4.5 (3.1-5.8)
Samoa	29.4 (24.9-33.9)	47.2 (41.0-53.3)	17.7 (14.2-21.3)	5.7 (2.8-8.6)	27.7 (24.0-31.4)	46.1 (42.6-49.6)	20.8 (18.6-23.0)	5.4 (3.4-7.4)
Solomon Islands	24.2 (14.3-34.2)	43.0 (33.2-52.7)	25.5 (20.1-30.9)	7.3 (2.1-12.6)	18.2 (13.7-22.8)	45.3 (38.2-52.5)	28.9 (21.4-36.4)	7.5 (4.7-10.4)
Tonga	27.1 (22.8-31.4)	43.7 (38.4-49.0)	22.2 (15.9-28.5)	7.0 (4.5-9.5)	29.4 (26.4-32.4)	43.7 (40.9-46.6)	19.4 (16.9-21.8)	7.5 (5.9-9.0)
Vanuatu	15.5 (2.4-28.7)	35.5 (26.9-44.2)	42.2 (25.4-59.1)	6.7 (1.3-12.1)	11.1 (4.6-17.7)	27.3 (18.6-36.0)	51.0 (38.1-63.9)	10.6 (5.5-15.7)
Vietnam*	9.2	47.2	30.6	12.9	6.5	33.5	49.4	10.7

					(4.8-8.1)	(30.1-36.9)	(44.7-54.0)	(9.0-12.4)
Wallis and Futuna	25.2	48.1	20.9	5.8	20.3	54.1	20.4	5.1
	(18.5-32.0)	(40.8-55.4)	(16.7-25.1)	(3.0-8.6)	(16.3-24.3)	(48.6-59.7)	(16.5-24.3)	(3.1-7.1)
**Pooled estimates**	**22.5**	**44.5**	**25.7**	**6.8**	**20.8**	**43.2**	**28.4**	**7.2**
	**(17.2-27.7)**	**(41.0-48.0)**	**(20.3-31.2)**	**(4.8-8.9)**	**(15.4-26.2)**	**(40.0-46.3)**	**(23.0-33.9)**	**(5.1-9.2)**
**I^2(%)**	95.9	87.6	95.4	89.6	98.0	90.6	97.5	95.8
**Total**								
**Pooled estimates***	**24.9**	**43.8**	**24.1**	**6.2**	**23.6**	**42.7**	**25.7**	**6.6**
	**(22.8-26.9)**	**(42.4-45.3)**	**(21.6-26.6)**	**(5.0-7.4)**	**(21.0-26.2)**	**(42.0-43.4)**	**(23.2-28.3)**	**(5.3-7.9)**
**I^2(%)**	0	0	33.7	45.9	0	0	22.0	53.9

*There was significantly difference in the prevalence of joint physical behavior between different age group (P<0.05).

Table S3. Combined physical behaviors among young adolescents aged 12-15 years by BMI, region, and country group.

	Normal weight, % (95% CI)				Overweight, % (95% CI)				Obesity, % (95% CI)			
	Low	Moderate-low	Moderate-high	High	Low	Moderate-low	Moderate-high	High	Low	Moderate-low	Moderate-high	High
**Africa Region**												
Algeria*	16.6	42.8	34.3	6.3	18.3	39.5	35.6	6.6	25.4	31.1	38.6	4.8
	(13.8-19.4)	(40.2-45.5)	(31.8-36.7)	(5.1-7.5)	(11.1-25.5)	(31.1-47.9)	(28.7-42.5)	(3.7-9.6)	(17.4-33.4)	(25.5-36.7)	(30.5-46.8)	(1.4-8.3)
Benin	5.2	28.2	42.3	24.3	4.5	34.3	39.7	21.5	6.0	20.9	55.5	17.6
	(3.2-7.2)	(24.0-32.4)	(38.7-45.9)	(20.4-28.1)	(0.0-9.0)	(21.9-46.7)	(32.7-46.7)	(12.3-30.7)	(2.8-9.3)	(11.3-30.4)	(45.9-65.0)	(13.6-21.6)
Mauritania*	28.9	38.9	24.8	7.4	33.3	38.9	20.8	7.0	23.5	48.8	21.2	6.5
	(23.7-34.2)	(32.9-44.8)	(19.7-29.9)	(3.8-10.9)	(21.0-45.5)	(28.1-49.7)	(10.6-31.1)	(2.0-12.0)	(2.8-44.2)	(32.6-65.1)	(7.7-34.7)	(0.0-15.5)
Mauritius*	20.7	47.1	26.7	5.5	18.5	41.1	33.3	7.0	14.3	46.7	33.0	6.1
	(16.2-25.1)	(43.5-50.7)	(23.2-30.3)	(3.8-7.2)	(10.4-26.6)	(35.2-47.0)	(26.9-39.8)	(2.8-11.2)	(6.6-22.0)	(34.7-58.6)	(22.3-43.7)	(2.5-9.6)
Mozambique*	30.4	51.5	18.1		36.0	45.0	19.0		40.2	48.1	11.7	
	(20.1-40.8)	(46.1-56.8)	(8.6-27.5)		(9.8-100.0)	(3.9-94.0)	(5.9-94.4)					
Namibia*	33.4	41.0	19.5	6.1	33.5	43.1	18.3	5.2	25.1	49.2	22.4	3.3
	(29.6-37.2)	(38.2-43.7)	(17.3-21.7)	(4.3-8.0)	(25.4-41.6)	(36.4-49.7)	(10.7-25.8)	(1.6-8.7)	(20.5-29.8)	(40.2-58.2)	(12.8-32.1)	(0.0-6.7)
Seychelles	28.1	43.8	22.1	6.0	27.0	47.4	22.0	3.7	33.4	47.9	16.6	2.1
	(25.0-31.2)	(40.8-46.8)	(19.3-24.9)	(4.6-7.5)	(18.6-35.3)	(38.5-56.2)	(14.9-29.1)	(0.3-7.1)	(22.9-43.9)	(36.5-59.2)	(6.9-26.3)	(0.0-5.1)
United Republic	18.7	41.5	25.0	14.8	17.3	35.9	38.6	8.2	16.2	47.5	23.2	13.1
of Tanzania*	(11.2-26.2)	(34.5-48.6)	(20.6-29.3)	(12.4-17.2)					(3.4-59.0)	(8.2-100.0)	(7.4-100.0)	(4.1-65.7)
**Pooled estimates**	**22.6**	**41.9**	**26.8**	**9.9**	**23.0**	**41.4**	**28.6**	**6.9**	**20.4**	**40.3**	**31.1**	**6.8**
	**(14.2-31.0)**	**(37.8-46.0)**	**(21.0-32.7)**	**(6.6-13.2)**	**(12.8-33.2)**	**(38.1-44.7)**	**(21.4-35.7)**	**(4.1-9.7)**	**(10.9-29.9)**	**(30.8-49.8)**	**(19.7-42.4)**	**(2.4-11.1)**
**I^2(%)**	97.5	89.2	95.8	94.4	89.9	0.0	82.1	62.4	91.0	80.3	85.7	86.4
**Region of the Americas**												

Antigua and	24.2	40.0	31.6	4.1	48.4	36.4	15.2	-	-	57.3	34.6	8.1
Barbuda*	(15.8-32.6)	(29.7-50.3)	(23.8-39.5)	(0.9-7.3)	(8.5-88.4)	(25.4-47.4)	(0.0-53.1)			(33.5-81.2)	(5.9-63.3)	(0.0-28.5)
Argentina*	9.7	42.0	38.4	9.8	12.2	36.2	42.4	9.2	15.2	36.4	35.9	12.5
	(8.5-10.9)	(39.5-44.6)	(36.1-40.7)	(8.6-11.1)	(8.0-16.3)	(31.9-40.6)	(37.5-47.3)	(7.1-11.2)	(9.9-20.5)	(28.9-43.9)	(28.2-43.5)	(4.8-20.2)
Barbados*	36.7	42.2	17.3	3.8	40.8	40.3	17.6	1.3	44.7	42.7	11.2	1.5
	(32.4-41.0)	(38.5-45.9)	(14.4-20.3)	(2.4-5.1)	(30.1-51.5)	(31.1-49.6)	(11.3-23.9)	(0.0-2.7)	(32.5-56.8)	(30.8-54.6)	(2.9-19.4)	(0.0-4.5)
Belize*	23.9	38.1	30.4	7.6	18.1	41.1	32.6	8.1	25.5	39.5	27.9	7.1
	(20.8-27.1)	(34.2-41.9)	(25.6-35.2)	(5.6-9.7)	(9.0-27.2)	(32.9-49.4)	(23.5-41.7)	(3.9-12.4)	(10.0-41.0)	(27.6-51.5)	(12.7-43.1)	(0.8-13.3)
Bolivia	16.5	41.4	32.9	9.2	9.8	48.5	35.2	6.6	17.5	38.3	39.1	5.2
	(14.0-19.0)	(39.0-43.7)	(29.9-36.0)	(7.5-11.0)	(5.7-13.8)	(38.4-58.5)	(27.4-43.1)	(4.0-9.2)	(13.1-21.8)	(30.9-45.6)	(33.2-45.0)	(2.5-7.9)
Chile	15.0	42.5	33.4	9.2	18.9	42.2	30.5	8.4	10.4	43.2	35.9	10.4
	(11.9-18.0)	(37.8-47.2)	(29.7-37.1)	(7.0-11.3)	(13.8-24.1)	(34.6-49.8)	(21.0-40.0)	(2.3-14.5)	(6.4-14.4)	(30.7-55.8)	(22.5-49.3)	(4.1-16.7)
Bahamas*	37.1	44.9	15.6	2.4	29.0	47.1	23.0	0.8	28.7	48.7	16.7	5.9
	(32.7-41.6)	(41.0-48.8)	(12.3-18.9)	(1.2-3.6)	(20.9-37.1)	(38.9-55.3)	(13.5-32.6)	(0.0-2.6)	(18.6-38.8)	(38.1-59.3)	(7.3-26.0)	(0.0-13.4)
Costa Rica*	19.2	41.2	30.3	9.4	21.6	39.5	29.9	9.1	22.4	42.6	28.1	6.8
	(15.2-23.1)	(38.1-44.3)	(27.6-32.9)	(7.8-11.0)	(13.1-30.0)	(31.7-47.2)	(21.9-37.9)	(5.0-13.1)	(14.9-29.9)	(36.9-48.4)	(19.5-36.8)	(0.0-14.4)
Curaçao*	31.5	45.8	19.1	3.6	42.3	37.2	14.8	5.6	25.7	44.8	17.8	11.8
	(27.3-35.7)	(42.1-49.5)	(14.5-23.7)	(1.9-5.3)	(30.4-54.2)	(27.3-47.2)	(4.8-24.8)	(0.8-10.5)	(10.8-40.6)	(27.6-61.9)	(1.9-33.7)	(1.8-21.7)
El Salvador*	18.8	42.9	30.9	7.4	15.9	45.1	30.9	8.1	23.2	40.2	27.0	9.6
	(14.8-22.8)	(40.5-45.3)	(26.9-34.9)	(5.2-9.7)	(10.7-21.1)	(36.0-54.2)	(19.1-42.6)	(3.8-12.5)	(14.4-31.9)	(26.7-53.6)	(16.5-37.6)	(4.4-14.9)
Guatemala	19.5	41.4	33.4	5.7	12.9	54.7	24.7	7.8	13.7	56.9	28.4	0.9
	(14.9-24.0)	(36.8-46.0)	(28.8-38.0)	(3.3-8.1)	(6.6-19.1)	(49.2-60.2)	(19.4-30.0)	(3.8-11.7)	(10.2-17.2)	(50.9-63.0)	(19.4-37.4)	(0.1-1.8)
Guyana*	34.0	46.4	16.6	3.1	35.7	46.1	14.3	3.9	43.8	36.2	15.3	4.7
	(28.7-39.3)	(43.4-49.3)	(12.1-21.0)	(1.9-4.3)	(25.5-45.9)	(37.3-54.8)	(8.3-20.3)	(0.7-7.1)	(28.7-58.8)	(26.4-46.0)	(7.6-23.0)	(0.1-9.3)
Honduras*	17.2	44.3	30.9	7.6	14.8	39.8	38.6	6.8	11.3	43.2	36.3	9.3
	(13.5-20.9)	(40.0-48.6)	(26.2-35.6)	(5.7-9.5)	(8.4-21.3)	(31.5-48.0)	(30.8-46.4)	(3.8-9.9)	(4.9-17.6)	(35.0-51.5)	(24.1-48.4)	(2.6-16.0)
Peru	17.1	43.6	34.1	5.2	15.9	46.4	32.0	5.7	21.5	42.1	31.9	4.4

	(13.7-20.5)	(41.2-46.1)	(30.3-37.9)	(3.8-6.6)	(10.7-21.1)	(39.8-53.0)	(23.6-40.4)	(2.7-8.6)	(13.1-30.0)	(32.0-52.2)	(22.0-41.8)	(2.2-6.7)
Suriname	23.4	43.0	27.6	5.9	22.5	35.9	29.0	12.5	21.9	39.8	33.9	4.3
	(17.9-29.0)	(39.1-47.0)	(22.3-33.0)	(4.5-7.3)	(12.0-33.0)	(29.3-42.5)	(21.2-36.9)	(3.6-21.5)	(9.8-34.1)	(30.2-49.4)	(25.8-42.0)	(0.0-11.3)
Trinidad and	29.0	46.3	20.3	4.4	32.4	44.9	20.0	2.7	30.8	44.7	17.6	7.0
Tobago*	(24.7-33.2)	(42.9-49.6)	(18.1-22.5)	(3.3-5.5)	(22.9-41.9)	(36.5-53.2)	(13.4-26.6)	(0.9-4.4)	(21.2-40.3)	(35.8-53.6)	(11.5-23.6)	(0.0-14.1)
Uruguay*	11.6	40.7	36.4	11.4	12.4	44.7	35.5	7.4	9.7	31.2	45.3	13.8
	(9.2-14.0)	(37.6-43.8)	(33.5-39.3)	(9.2-13.6)	(8.8-16.0)	(38.0-51.4)	(27.7-43.3)	(3.3-11.5)	(4.7-14.7)	(24.3-38.0)	(37.4-53.3)	(9.1-18.5)
**Pooled estimates**	**22.5**	**42.9**	**28.2**	**6.4**	**21.2**	**42.8**	**27.9**	**6.4**	**21.2**	**40.4**	**28.1**	**6.5**
	**(18.2-26.7)**	**(41.8-43.9)**	**(24.3-32.1)**	**(5.1-7.8)**	**(17.1-25.2)**	**(39.9-45.7)**	**(23.3-32.5)**	**(4.4-8.3)**	**(17.2-25.2)**	**(36.0-44.8)**	**(22.7-33.4)**	**(4.3-8.6)**
**I^2(%)**	96.6	37.1	95.3	91.5	83.8	60.0	83.3	86.5	80.0	73.9	82.7	80.6
**Eastern Mediterranean Region**												
Afghanistan	13.2	40.9	37.7	8.2	17.2	46.5	26.5	9.8	17.3	48.7	22.6	11.4
	(9.3-17.2)	(33.3-48.6)	(27.4-48.0)	(5.3-11.0)	(8.8-25.7)	(37.5-55.5)	(17.7-35.2)	(3.5-16.1)	(8.8-25.8)	(37.0-60.4)	(14.1-31.1)	(4.9-17.8)
Egypt	21.4	47.0	27.2	4.4	32.3	43.4	22.7	1.6	26.0	44.1	25.7	4.2
	(16.6-26.1)	(40.5-53.5)	(19.6-34.8)	(2.6-6.3)	(25.2-39.3)	(37.7-49.1)	(16.6-28.8)	(0.0-3.5)	(17.1-34.9)	(32.7-55.5)	(13.7-37.8)	(0.7-7.6)
Iraq*	25.4	39.1	27.5	8.0	31.2	34.8	24.4	9.6	35.8	36.7	20.2	7.3
	(21.4-29.4)	(35.3-43.0)	(23.5-31.5)	(5.8-10.2)	(22.4-40.0)	(25.6-44.0)	(14.3-34.6)	(5.3-13.8)	(26.9-44.8)	(25.5-48.0)	(12.6-27.7)	(0.0-15.0)
Kuwait*	31.7	50.4	14.7	3.2	35.1	50.3	13.1	1.5	43.0	51.6	3.4	2.0
	(26.3-37.0)	(45.3-55.5)	(10.7-18.8)	(1.6-4.8)	(25.2-44.9)	(39.9-60.7)	(7.2-19.0)	(0.0-4.0)	(33.0-53.0)	(41.9-61.3)	(0.7-6.1)	(0.0-4.5)
Lebanon*	26.7	47.4	23.1	2.8	25.3	51.0	21.9	1.8	21.5	52.6	23.0	2.9
	(22.4-31.0)	(42.4-52.4)	(16.3-29.9)	(2.0-3.6)	(15.9-34.8)	(41.8-60.1)	(13.0-30.7)	(0.4-3.2)	(12.6-30.4)	(41.4-63.8)	(14.8-31.1)	(0.0-6.6)
Morocco*	15.1	37.2	37.8	9.9	12.9	35.7	40.7	10.7	11.2	32.3	45.5	11.0
	(12.9-17.2)	(33.7-40.7)	(33.8-41.8)	(8.3-11.6)	(8.9-16.9)	(28.9-42.5)	(32.0-49.5)	(7.0-14.4)	(5.9-16.5)	(25.5-39.2)	(37.2-53.8)	(5.3-16.7)
Oman*	23.4	52.7	20.6	3.2	29.1	47.1	19.1	4.7	25.2	50.7	22.0	2.1
	(19.8-27.0)	(50.0-55.5)	(17.7-23.6)	(2.2-4.2)	(21.7-36.5)	(39.2-55.0)	(12.0-26.3)	(1.9-7.5)	(19.2-31.1)	(40.8-60.6)	(13.1-30.9)	(0.0-4.2)

Pakistan*	27.6 (20.8-34.4)	42.1 (39.2-45.1)	23.1 (18.5-27.7)	7.2 (3.2-11.2)	32.6 (21.9-43.4)	38.9 (32.5-45.4)	20.7 (14.6-26.7)	7.7 (4.0-11.4)	25.7 (16.2-35.1)	46.5 (37.3-55.8)	22.5 (18.0-27.0)	5.3 (2.8-7.8)
Qatar	27.0 (21.5-32.6)	44.6 (38.8-50.5)	19.7 (15.9-23.6)	8.6 (4.8-12.3)	33.7 (21.5-45.8)	48.1 (37.0-59.1)	15.3 (8.6-22.0)	3.0 (0.0-6.4)	25.7 (20.2-31.2)	56.8 (52.2-61.3)	14.5 (6.0-22.9)	3.1 (2.7-3.5)
Sudan*	37.6 (31.8-43.4)	46.9 (42.1-51.6)	13.5 (9.3-17.6)	2.1 (1.1-3.1)	44.3 (33.1-55.4)	41.1 (31.3-50.9)	13.0 (8.0-17.9)	1.7 (0.0-3.3)	31.9 (21.5-42.3)	50.1 (33.9-66.3)	17.1 (11.2-23.0)	0.9 (0.6-1.2)
Syrian Arab Republic	26.7 (22.4-31.0)	43.1 (39.4-46.7)	24.7 (21.4-27.9)	5.6 (4.0-7.2)	28.0 (20.9-35.1)	43.3 (36.0-50.6)	23.3 (17.9-28.6)	5.5 (1.8-9.1)	31.6 (22.2--41.1)	40.8 (32.1-49.5)	24.3 (14.6-34.0)	3.3 (0.0-7.6)
United Arab Emirates*	33.6 (28.4-38.9)	49.2 (45.2-53.2)	15.3 (12.7-17.9)	1.9 (0.9-2.8)	34.0 (26.4-41.6)	47.4 (39.9-54.9)	16.7 (11.2-22.3)	1.8 (0.1-3.6)	40.9 (30.8-50.9)	43.0 (33.7-52.3)	16.1 (8.6-23.6)	-
**Pooled estimates**	**25.6 (21.3-29.9)**	**45.1 (41.9-48.2)**	**23.3 (19.1-27.5)**	**5.1 (3.7-6.5)**	**29.2 (23.4-35.0)**	**43.5 (40.5-46.4)**	**20.9 (17.1-24.7)**	**4.2 (2.7-5.8)**	**27.5 (22.1-33.0)**	**46.1 (40.8-51.4)**	**21.2 (14.2-28.2)**	**3.5 (2.2-4.8)**
**I^2(%)**	91.9	85.2	91.9	91.1	84.5	38.1	74.4	78.5	82.0	75.1	92.7	90.6
**Southeast Asia Region**												
Bangladesh	9.9 (7.1-12.7)	22.0 (18.7-25.3)	32.9 (28.5-37.4)	35.1 (29.0-41.2)	12.9 (5.8-20.0)	19.6 (7.3-31.9)	38.9 (31.9-45.9)	28.6 (16.2-41.0)	3.5 (0.0-7.1)	17.5 (12.2-22.8)	59.3 (47.8-70.9)	19.7 (11.3-28.1)
Indonesia*	32.4 (29.1-35.7)	41.8 (39.9-43.7)	21.2 (18.1-24.4)	4.6 (3.4-5.8)	29.3 (25.0-33.5)	44.0 (38.7-49.3)	20.8 (16.6-25.1)	5.9 (3.5-8.3)	30.8 (24.6-37.0)	38.8 (32.6-45.0)	24.7 (19.2-30.1)	5.7 (2.8-8.7)
Thailand	31.3 (27.8-34.9)	48.1 (45.7-50.5)	16.8 (14.8-18.7)	3.8 (2.6-5.1)	37.1 (31.0-43.3)	43.9 (38.4-49.5)	16.6 (12.5-20.6)	2.4 (0.5-4.2)	33.9 (24.5-43.4)	44.1 (33.3-54.8)	20.3 (8.8-31.9)	1.7 (1.3-2.1)
Timor-Leste*	29.1 (26.3-31.9)	52.9 (50.9-54.9)	14.8 (12.8-16.8)	3.2 (2.2-4.3)	36.9 (30.3-43.5)	43.2 (36.2-50.2)	17.3 (9.0-25.5)	2.6 (1.5-3.8)	23.3 (17.9-28.8)	59.6 (50.9-68.2)	14.4 (6.2-22.7)	2.7 (0.0-6.5)
**Pooled estimates**	**25.7 (14.7-36.6)**	**41.3 (30.5-52.0)**	**21.1 (15.3-26.9)**	**9.4 (5.2-13.7)**	**29.2 (19.6-38.7)**	**39.5 (32.1-46.9)**	**23.2 (14.5-31.9)**	**5.0 (1.8-8.2)**	**22.6 (7.2-38.0)**	**39.8 (21.4-58.1)**	**29.3 (13.3-45.3)**	**5.5 (1.5-9.5)**
**I^2(%)**	98.0	98.9	94.9	97.1	90.5	78.3	90.1	86.8	96.6	96.1	92.6	87.8
**Western Pacific**												

Region												
Brunei Darussalam*	33.5	49.6	14.8	2.2	37.8	49.3	12.4	0.6	35.1	52.7	12.2	
	(30.6-36.4)	(46.4-52.7)	(12.3-17.2)	(1.3-3.0)	(31.0-44.6)	(42.8-55.7)	(7.1-17.6)	(0.0-1.8)	(26.6-43.6)	(42.7-62.7)	(7.1-17.3)	
Cambodia*	19.8	42.4	31.9	5.9	16.4	49.9	30.6	3.1	29.4	31.6	34.6	4.4
	(16.3-23.4)	(39.2-45.6)	(27.0-36.8)	(4.8-7.0)	(9.4-23.4)	(43.2-56.7)	(23.2-38.1)	(0.9-5.2)	(15.9-42.9)	(22.2-41.1)	(23.8-45.4)	(0.5-8.3)
Kiribati	18.8	44.1	25.9	11.2	27.3	39.8	22.5	10.3	17.6	50.5	26.0	5.8
	(16.0-21.7)	(39.3-48.9)	(22.3-29.4)	(8.2-14.2)	(20.5-34.2)	(33.2-46.4)	(14.7-30.3)	(4.2-16.5)	(8.3-27.0)	(35.9-65.2)	(14.6-37.4)	(0.6-11.1)
Lao People's Democratic Republic*	25.6	49.1	20.8	4.5	24.8	47.8	23.0	4.4	31.7	47.6	17.3	3.4
	(21.2-30.0)	(46.0-52.2)	(16.4-25.1)	(3.1-6.0)	(17.3-32.3)	(40.6-55.1)	(15.3-30.6)	(1.5-7.3)	(22.0-41.4)	(42.4-52.7)	(9.3-25.4)	(0.0-7.5)
Malaysia*	21.7	45.7	25.5	7.0	21.0	46.7	24.6	7.7	23.4	43.9	27.0	5.7
	(19.9-23.6)	(44.1-47.4)	(23.6-27.5)	(6.1-7.9)	(18.2-23.9)	(43.2-50.2)	(21.3-28.0)	(5.8-9.5)	(19.7-27.1)	(38.9-48.9)	(22.7-31.3)	(3.3-8.1)
Mongolia	7.3	31.7	45.3	15.8	12.1	31.4	44.5	12.0	11.6	38.2	41.8	8.3
	(6.0-8.6)	(28.8-34.7)	(42.8-47.7)	(13.5-18.0)	(7.8-16.5)	(26.3-36.5)	(39.1-49.8)	(8.4-15.6)	(7.6-15.7)	(30.5-46.0)	(33.1-50.6)	(4.8-11.9)
Philippines	23.1	47.0	25.4	4.5	25.2	44.4	26.7	3.7	32.3	44.4	17.7	5.6
	(20.4-25.8)	(44.7-49.3)	(23.0-27.8)	(3.5-5.5)	(18.4-31.9)	(40.7-48.1)	(21.1-32.2)	(1.6-5.8)	(22.4-42.2)	(39.0-49.8)	(10.5-24.9)	(1.7-9.5)
Samoa	28.6	46.7	18.3	6.4	26.3	53.4	17.2	3.1	33.6	47.4	19.0	
	(23.8-33.3)	(42.7-50.8)	(15.6-21.1)	(4.6-8.1)	(16.5-36.1)	(41.4-65.5)	(12.0-22.4)	(0.3-5.9)	(22.8-44.4)	(37.1-57.7)	(12.6-25.4)	
Solomon Islands	21.7	44.2	26.2	7.8	8.0	53.7	27.7	10.6	16.2	36.8	45.1	1.9
	(14.5-29.0)	(36.6-51.9)	(18.0-34.4)	(3.1-12.6)	(0.5-15.6)	(41.0-66.5)	(16.6-38.7)	(6.0-15.1)	(6.4-26.0)	(18.5-55.0)	(27.6-62.6)	(0.0-6.4)
Tonga	27.6	44.1	20.6	7.7	29.1	44.4	20.3	6.2	35.1	40.6	16.0	8.2
	(24.7-30.6)	(41.3-46.9)	(17.8-23.4)	(6.1-9.3)	(22.5-35.6)	(37.0-51.7)	(14.1-26.6)	(2.1-10.4)	(23.9-46.4)	(29.9-51.3)	(8.9-23.2)	(2.6-13.9)
Vanuatu	17.4	29.4	45.6	7.6	13.7	34.0	36.9	15.4	7.3	33.7	55.3	3.7
	(10.9-24.0)	(19.3-39.6)	(32.2-59.0)	(2.1-13.0)	(3.9-23.4)	(9.7-58.3)	(27.0-46.8)	(0.0-37.7)	(5.8-8.9)	(17.4-50.0)	(31.6-78.9)	(0.0-14.5)
Vietnam*	5.8	34.3	49.6	10.3	10.5	29.6	51.7	8.1	7.8	42.4	38.5	11.3
	(4.1-7.5)	(31.0-37.6)	(45.3-53.8)	(8.1-12.5)	(5.9-15.1)	(20.3-38.9)	(42.6-60.9)	(4.6-11.7)	(0.2-15.4)	(26.8-58.1)	(23.9-53.1)	(6.4-16.1)
Wallis and Futuna	23.0	50.7	20.7	5.6	20.9	48.4	20.2	10.5	18.3	64.1	17.6	

	(19.1-26.8)	(46.1-55.3)	(17.6-23.8)	(3.7-7.5)	(11.0-30.9)	(34.6-62.2)	(11.3-29.1)	(2.2-18.8)	(4.3-32.3)	(46.8-81.4)	(6.4-28.7)	
**Pooled estimates**	**21.0**	**43.4**	**28.1**	**7.3**	**20.9**	**44.1**	**27.3**	**6.3**	**22.5**	**43.9**	**25.9**	**5.6**
	**(15.6-26.5)**	**(40.2-46.6)**	**(22.3-33.9)**	**(5.5-9.0)**	**(16.4-25.4)**	**(40.1-48.0)**	**(21.6-33.0)**	**(4.0-8.7)**	**(16.2-28.8)**	**(40.4-47.5)**	**(20.3-31.6)**	**(4.0-7.3)**
**I^2(%)**	98.1	92.1	97.7	93.9	85.9	75.4	90.5	88.9	93.1	46.6	83.0	41.6
**Total**												
**Pooled estimates***	**23.4**	**43.1**	**25.6**	**7.0**	**23.9**	**42.7**	**25.3**	**5.6**	**23.0**	**43.0**	**26.3**	**5.2**
	**(20.96-25.8)**	**(42.1-44.0)**	**(22.9-28.3)**	**(5.5-8.4)**	**(20.2-27.7)**	**(41.2-44.3)**	**(21.9-28.8)**	**(4.5-6.7)**	**(20.3-25.7)**	**(40.7-45.4)**	**(23.1-29.4)**	**(3.8-6.6)**
**I^2(%)**	0	0	33.8	62.0	47.1	0	48.3	20.3	0	0	0	50.4

*There was significantly difference in the prevalence of joint physical behavior between different BMI group (*P*<0.05).

Table S4. The percentage of physically active for at least 60 min among young adolescents aged 12-15 years.

	Percentage of physically active adolescents on 7 days per week (95% CI)							
	**Overall**	**Boys, %**	**Girls, %**	**12-13, %**	**14-15, %**	**Normal weight, %**	**Overweight, %**	**Obesity, %**
**Africa Region**								
Algeria*	15.0 (13.5-16.6)	23.7 (20.4-27.1) *	7.7 (6.1-9.2)	15.1 (13.1-17.1)	14.8 (12.6-17.1)	15.4 (13.7-17.1)	15.0 (9.0-20.9)	14.2 (6.5-22.0)
Benin	24.4 (20.7-28.0)	27.3 (23.4-31.1) *	19.3 (14.1-24.6)	26.4 (22.2-30.5)	23.9 (19.9-27.9)	25.6 (21.7-29.5)	21.7 (14.1-29.3)	19.2 (13.8-24.6)
Mauritania*	10.5 (7.9-13.2)	13.9 (9.5-18.3) *	6.8 (4.7-8.9)	9.3 (5.3-13.2)	11.1 (8.1-14.1)	10.6 (6.4-14.8)	6.4 (0.2-12.5)	6.5 (0.0-15.5)
Mauritius*	19.4 (15.4-23.4)	26.0 (21.3-30.7) *	13.3 (10.8-15.7)	18.1 (13.9-22.3)	20.2 (15.6-24.8)	19.6 (15.6-23.7)	20.6 (15.3-25.9)	42.0 (29.3-54.7)
Mozambique*	11.0 (4.4-17.7)	12.6 (7.2-18.0) *	8.7 (0.0-17.6)	13.7 (9.3-18.0)	10.1 (2.6-17.5)	8.3 (0.0-18.1)	7.4 (0.0-94.5)	-
Namibia*	13.8 (11.5-16.1)	14.5 (11.6-17.4)	13.3 (10.6-15.9)	13.8 (10.4-17.3)	13.7 (11.5-15.8)	13.8 (11.3-16.2)	13.8 (6.2-21.5)	13.3 (4.0-22.6)
Seychelles	17.5 (15.5-19.4)	19.8 (16.3-23.3) *	15.4 (12.8-18.0)	16.1 (13.1-19.1)	18.7 (16.2-21.3)	19.1 (16.8-21.4) *	14.5 (8.9-20.1)	12.3 (4.1-20.6)
United Republic of Tanzania*	20.7 (17.0-24.3)	23.6 (17.8-29.4) *	18.0 (14.6-21.4)	19.8 (16.4-23.2)	21.3 (15.8-26.9)	24.5 (18.0-31.1)	-	17.1 (0-100.0)
**Pooled estimates**	**16.6 (13.9-19.3)**	**20.2 (16.2-24.2)**	**12.8 (9.6-16.0)**	**16.5 (13.5-19.5)**	**16.7 (13.58-19.7)**	**17.4 (14.2-20.5)**	**15.2 (11.0-19.5)**	**17.0 (9.9-24.0)**
**I^2(%)**	87.4	87.2	90.8	84.2	84.1	86.2	59.9	75.5
**Region of the Americas**								

Antigua and	22.4	28.4	16.4	20.4	23.6	26.8	15.1	49.4
Barbuda*	(18.5-26.4)	(22.3-34.6) *	(12.4-20.3)	(15.8-25.0)	(18.5-28.6)	(18.5-35.1) *	(13.3-16.9)	(14.7-84.0)
Argentina*	16.4	21.2	12.1	17.0	16.2	17.3	18.1	17.1
	(15.2-17.6)	(19.7-22.7) *	(10.9-13.4)	(14.4-19.7)	(15.1-17.3)	(15.7-18.9)	(15.0-21.2)	(12.3-21.8)
Barbados*	19.4	25.0	13.7	17.5	19.9	20.9	10.7	14.8
	(16.9-21.9)	(21.1-28.9) *	(10.5-16.8)	(12.6-22.4)	(16.7-23.0)	(18.0-23.8) *	(4.9-16.6)	(7.7-21.8)
Belize*	20.0	23.6	16.7	20.8	19.4	21.2	16.5	19.0
	(17.3-22.8)	(20.3-26.9) *	(14.2-19.2)	(17.5-24.2)	(15.9-22.9)	(18.0-24.4)	(8.9-24.0)	(4.4-33.6)
Bolivia	13.4	16.5	11.2	14.0	13.7	14.1	13.7	12.4
	(11.7-15.2)	(14.1-18.9) *	(8.6-13.9)	(10.8-17.2)	(11.6-15.8)	(12.2-15.9)	(7.9-19.5)	(7.9-16.8)
Chile	15.5	22.5	8.7	17.1	14.5	16.3	11.4	12.9
	(13.1-17.9)	(18.4-26.6) *	(5.9-11.4)	(13.7-20.5)	(11.5-17.5)	(13.3-19.3)	(6.0-16.9)	(4.9-20.8)
Bahamas*	15.5	19.6	11.9	15.0	16.3	15.5	11.9	18.7
	(12.0-19.0)	(13.4-25.8) *	(9.3-14.5)	(11.3-18.6)	(10.8-21.8)	(12.1-19.0)	(5.8-18.0)	(8.5-29.0)
Costa Rica*	18.0	24.7	11.4	19.0	17.5	18.7	15.5	15.6
	(15.8-20.1)	(21.9-27.6) *	(8.8-13.9)	(16.3-21.7)	(14.7-20.3)	(16.3-21.1)	(10.2-20.8)	(9.7-21.5)
Curaçao*	11.6	15.5	7.9	11.5	11.6	10.7	11.3	18.5
	(10.0-13.2)	(12.9-18.1) *	(6.0-9.8)	(8.4-14.6)	(9.6-13.5)	(8.6-12.8)	(3.8-18.8)	(3.3-33.6)
El Salvador*	12.5	15.9	9.1	8.7	14.2	12.3	16.1	11.4
	(9.9-15.0)	(12.5-19.3) *	(6.7-11.5)	(5.1-12.3) *	(11.7-16.7)	(9.3-15.2)	(11.2-21.1)	(5.6-17.3)
Guatemala	11.1	12.8	9.7	12.3	10.8	11.6	8.0	3.1
	(7.8-14.5)	(8.2-17.4)	(6.8-12.5)	(9.8-14.7)	(6.5-15.2)	(8.4-14.9) *	(1.4-14.6)	(1.6-4.7)
Guyana*	14.9	18.4	12.1	13.6	15.6	15.8	13.9	15.3
	(11.1-18.8)	(12.6-24.2) *	(8.9-15.2)	(6.2-20.9)	(12.5-18.6)	(11.7-19.8)	(8.1-19.7)	(6.0-24.6)
Honduras*	15.1	18.1	12.6	14.6	15.8	15.1	18.5	16.2
	(12.7-17.6)	(15.2-21.1) *	(9.5-15.7)	(9.8-19.4)	(12.8-18.7)	(11.5-18.7)	(10.7-26.3)	(3.5-28.8)
Peru	15.2	17.1	13.3	18.1	14.1	15.3	13.2	13.2

	(13.2-17.2)	(14.4-19.9) *	(11.1-15.5)	(14.1-22.0) *	(12.5-15.8)	(13.2-17.4)	(8.0-18.3)	(7.5-19.0)
Suriname	20.2	24.8	16.2	21.2	19.6	20.7	20.0	15.3
	(16.7-23.7)	(20.0-29.6) *	(13.1-19.4)	(14.9-27.6)	(16.0-23.1)	(17.8-23.7)	(10.0-30.0)	(4.4-26.2)
Trinidad and	20.2	25.0	15.8	20.7	20.0	21.4	12.9	15.8
Tobago*	(17.1-23.3)	(21.5-28.6) *	(13.0-18.5)	(17.5-24.0)	(16.4-23.6)	(17.7-25.1) *	(6.4-19.4)	(4.1-27.5)
Uruguay*	16.1	23.6	9.6	16.3	16.0	16.4	13.8	16.1
	(14.2-18.0)	(20.9-26.4) *	(8.0-11.2)	(13.2-19.4)	(14.0-18.1)	(14.5-18.3)	(10.1-17.6)	(7.1-25.2)
**Pooled estimates**	**16.2**	**20.6**	**12.1**	**16.3**	**16.1**	**16.7**	**14.6**	**14.5**
	**(14.2-18.0)**	**(18.7-22.5)**	**(10.8-13.4)**	**(14.5-18.0)**	**(14.8-17.4)**	**(15.1-18.3)**	**(13.4-15.8)**	**(10.6-18.5)**
**I^2(%)**	83.2	82.3	78.6	75.1	75.0	83.2	7.0	83.4
**Eastern Mediterranean Region**								
Afghanistan	8.8	7.5	11.1	9.5	8.9	8.3	8.9	9.9
	(5.8-11.9)	(3.4-11.6) *	(9.0-13.2)	(5.1-13.9)	(5.7-12.1)	(5.0-11.7)	(4.7-13.0)	(1.5-18.4)
Egypt	12.6	18.6	7.0	13.5	11.6	13.2	8.2	13.8
	(8.9-16.3)	(13.5-23.7) *	(4.7-9.3)	(7.8-19.1)	(8.3-14.8)	(9.4-17.1)	(3.9-12.6)	(6.5-21.0)
Iraq*	14.7	19.5	8.9	16.8	13.7	14.7	14.6	16.2
	(12.3-17.1)	(16.6-22.4) *	(5.5-12.4)	(12.8-20.8)	(11.2-16.3)	(12.3-17.1)	(9.2-20.0)	(7.2-25.1)
Kuwait*	16.8	18.9	14.7	19.1	15.9	17.3	15.6	11.9
	(14.1-19.5)	(15.7-22.2) *	(12.8-16.5)	(15.3-22.8)	(13.2-18.6)	(14.2-20.4)	(9.0-22.3)	(5.4-18.5)
Lebanon*	23.4	31.2	16.6	26.0	21.8	24.2	26.2	23.4
	(20.6-26.3)	(26.1-36.4) *	(14.5-18.8)	(22.8-29.3)	(19.1-24.5)	(20.1-28.2)	(19.7-32.7)	(14.9-31.9)
Morocco*	12.4	14.7	10.1	11.9	12.8	12.9	13.0	12.3
	(11.1-13.7)	(13.1-16.2) *	(7.8-12.4)	(9.8-14.0)	(11.6-14.0)	(11.9-13.9)	(7.4-18.5)	(6.9-17.7)
Oman*	13.0	16.4	10.1	14.2	12.6	13.2	14.8	15.0
	(10.5-15.5)	(14.0-18.8) *	(7.8-12.3)	(10.9-17.6)	(9.8-15.4)	(10.1-16.3)	(7.5-22.2)	(9.6-20.5)

Pakistan*	11.6	12.9	9.4	11.9	11.4	11.5	12.8	9.5
	(5.9-17.3)	(6.4-19.4)	(5.5-13.3)	(3.0-20.9)	(6.1-16.8)	(5.8-17.1)	(6.9-18.7)	(3.7-15.3)
Qatar	10.5	12.8	8.6	12.7	8.1	13.4	12.0	5.9
	(7.3-13.6)	(8.4-17.2)	(4.0-13.2)	(8.2-17.2)	(4.8-11.5)	(7.0-19.8)	(5.5-18.6)	(5.1-6.7)
Sudan*	7.8	8.2	7.4	8.9	7.3	7.9	7.9	9.4
	(5.5-10.1)	(4.5-11.9)	(4.4-10.4)	(2.9-14.9)	(4.6-10.0)	(5.3-10.6)	(4.9-10.8)	(5.6-13.2)
Syrian Arab	11.2	14.7	7.6	10.6	11.8	12.0	12.0	6.2
Republic	(9.2-13.2)	(11.9-17.4) *	(5.7-9.6)	(8.3-13.0)	(9.2-14.5)	(9.6-14.3)	(8.1-15.9)	(1.4-11.0)
United Arab	17.1	22.7	13.5	18.8	16.3	18.5	14.0	10.1
Emirates*	(14.9-19.4)	(19.4-25.9) *	(11.1-16.0)	(16.2-21.4)	(13.8-18.8)	(15.9-21.2)	(8.5-19.5)	(5.3-14.9)
**Pooled estimates**	**13.4**	**16.5**	**10.5**	**14.7**	**12.8**	**13.9**	**12.8**	**11.2**
	**(11.1-15.6)**	**(13.7-19.3)**	**(8.6-12.4)**	**(11.7-17.7)**	**(10.7-14.9)**	**(11.8-16.0)**	**(10.3-15.4)**	**(8.4-14.0)**
**I^2(%)**	89.7	89.0	86.0	87.9	87.2	85.7	67.5	76.8
**Southeast Asia Region**								
Bangladesh	42.2	42.4	41.9	34.0	45.7	43.6	36.8	40.7
	(37.0-47.4)	(36.1-48.6)	(34.6-49.1)	(27.9-40.1) *	(39.5-51.9)	(38.4-48.8)	(30.2-43.4)	(27.1-54.3)
Indonesia*	11.9	12.7	11.3	11.4	12.5	11.8	13.7	13.9
	(10.5-13.4)	(10.8-14.5)	(9.5-13.1)	(9.4-13.5)	(10.6-14.5)	(10.4-13.2)	(11.4-15.9)	(10.2-17.6)
Thailand	11.9	17.3	6.8	12.2	11.7	12.2	9.9	10.0
	(10.1-13.7)	(13.9-20.7) *	(5.2-8.4)	(10.3-14.0)	(9.2-14.3)	(10.3-14.2)	(5.2-14.5)	(3.8-16.2)
Timor-Leste*	8.5	10.9	5.8	8.6	8.1	8.0	8.1	8.6
	(6.4-10.5)	(8.1-13.6) *	(4.2-7.5)	(5.7-11.4)	(6.1-10.1)	(5.7-10.4)	(4.2-12.0)	(1.7-15.5)
**Pooled estimates**	**18.0**	**20.2**	**14.7**	**15.6**	**18.8**	**18.4**	**16.7**	**16.0**
	**(10.8-25.1)**	**(11.9-28.5)**	**(8.5-20.9)**	**(9.9-21.3)**	**(10.4-27.1)**	**(10.5-26.2)**	**(8.0-25.4)**	**(7.8-24.2)**
**I^2(%)**	97.9	96.6	97.2	94.6	97.7	98.0	94.8	83.8
**Western Pacific**								

**Region**								
Brunei	11.5	18.4	5.0	10.7	11.8	12.6	8.8	3.4
Darussalam*	(10.1-12.8)	(16.0-20.9) *	(3.5-6.6)	(8.8-12.7)	(10.0-13.6)	(11.0-14.3)	(4.2-13.4)	(0.1-6.6)
Cambodia*	6.3	8.2	4.7	6.3	6.4	6.0	3.9	4.9
	(4.5-8.2)	(6.1-10.3)	(2.0-7.3)	(3.0-9.6)	(4.5-8.4)	(4.4-7.6)	(1.3-6.5)	(0.6-9.2)
Kiribati	17.1	20.7	14.2	14.6	18.1	16.9	20.3	12.0
	(15.0-19.1)	(16.6-24.7) *	(11.9-16.6)	(10.7-18.4)	(16.1-20.1)	(14.2-19.7)	(13.1-27.7)	(3.2-20.8)
Lao People's	16.2	24.6	8.8	17.0	16.2	16.2	15.0	16.1
Democratic	(13.6-18.9)	(20.3-28.9) *	(6.3-11.3)	(9.1-24.9)	(13.3-19.0)	(13.1-19.3)	(10.3-19.6)	(9.8-22.4)
Republic*								
Malaysia*	13.8	19.4	8.3	13.6	13.9	14.4	12.9	10.6
	(12.8-14.8)	(18.0-20.8) *	(7.3-9.2)	(12.2-14.9)	(12.5-15.3)	(13.3-15.6)	(10.4-15.3)	(8.0-13.3)
Mongolia	26.9	31.5	22.4	28.5	25.5	28.0	22.0	21.1
	(25.3-28.4)	(28.8-34.1) *	(20.1-24.7)	(25.5-31.6)	(23.3-27.5)	(26.0-30.0)	(16.7-27.3)	(14.8-27.5)
Philippines	7.3	7.8	6.9	7.7	7.2	7.3	10.5	11.5
	(5.6-9.1)	(5.5-10.2)	(4.9-8.9)	(6.0-9.4)	(5.0-9.4)	(5.5-9.0)	(5.4-15.5)	(7.1-16.0)
Samoa	12.2	10.9	13.5	10.5	12.7	13.3	9.3	7.1
	(10.5-13.8)	(7.9-13.9)	(10.6-16.4)	(7.3-13.7)	(10.4-15.0)	(10.6-16.0)	(3.9-14.8)	(0.0-15.9)
Solomon Islands	16.2	18.4	14.5	12.7	17.6	16.5	15.4	14.4
	(12.0-20.5)	(11.7-25.0)	(10.6-18.4)	(5.8-19.5) *	(13.9-21.3)	(12.0-20.9)	(8.3-22.5)	(1.1-27.7)
Tonga	13.8	12.0	15.5	13.5	13.9	14.3	13.2	13.2
	(11.7-15.9)	(9.7-14.4)	(12.6-18.5)	(9.9-17.1)	(11.7-16.0)	(12.1-16.5)	(7.9-18.5)	(6.0-20.3)
Vanuatu	10.7	12.4	9.4	7.4	14.2	8.9	9.0	4.9
	(6.5-14.8)	(6.7-18.0)	(5.3-13.5)	(1.3-13.5) *	(10.2-18.2)	(5.6-12.2)	(0.5-17.6)	(3.8-5.9)
Vietnam*	13.1	17.0	9.5	-	13.0	12.6	13.4	15.9
	(11.3-14.9)	(14.6-19.4) *	(7.4-11.7)		(11.4-14.7)	(10.5-14.7)	(9.7-17.1)	(9.9-21.8)
Wallis and Futuna	14.4	14.0	14.7	15.5	13.9	14.7	16.7	12.4

	(10.7-18.2)	(8.7-19.4)	(10.4-19.0)	(10.3-20.6)	(9.2-18.6)	(10.6-18.9)	(6.1-27.2)	(1.8-22.9)
**Pooled estimates**	**13.8**	**16.5**	**11.3**	**13.1**	**14.2**	**13.9**	**12.8**	**10.7**
	**(10.7-16.9)**	**(12.7-20.4)**	**(8.6-13.9)**	**(9.8-16.4)**	**(11.4-16.9)**	**(10.7-17.2)**	**(9.8-15.8)**	**(7.7-13.7)**
**I^2(%)**	97.2	96.0	94.8	93.3	95.0	96.6	80.9	83.6
**Total**								
**Pooled estimates***	**15.2**	**18.7**	**11.7**	**15.6**	**15.1**	**15.6**	**14.2**	**12.5**
	**(13.7-16.7)**	**(16.6-20.9)***	**(10.8-12.7)**	**(14.4-16.8)**	**(13.4-16.8)**	**(14.0-17.3)**	**(13.2-15.2)**	**(10.4-14.6)**
**I^2(%)**	32.5	48.8	0	0	55.5	43.4	0	25.3

*There was significantly difference between different sex, age, and BMI group (*P*<0.05).

Table S5. Percentage of engaged in active transportation among young adolescents aged 12-15 years walked or rode a bicycle to and from school at least 3 days during the past 7 days.

	Percentage of active transport on 7 days per week (95% CI)							
	**Overall**	**Boys, %**	**Girls, %**	**12-13, %**	**14-15, %**	**Normal weight, %**	**Overweight, %**	**Obesity, %**
**Africa Region**								
Algeria*	49.5	46.2	52.3	43.3	54.4	49.2	49.6	50.9
	(44.8-54.2)	(41.3-51.1) *	(47.2-57.5)	(37.2-49.5) *	(49.8-59.0)	(44.8-53.6)	(37.6-61.6)	(42.7-59.0)
Benin	77.5	77.4	78.4	73.5	78.7	78.3	75.1	73.1
	(73.7-81.2)	(74.8-80.0)	(70.6-86.2)	(66.9-80.2)	(74.6-82.8)	(74.6-82.0)	(64.1-86.1)	(62.5-83.6)
Mauritania*	51.2	55.1	47.4	49.5	51.8	50.3	45.9	56.5
	(46.7-55.8)	(50.8-59.4) *	(39.4-55.4)	(44.6-54.4)	(46.6-56.9)	(45.6-55.1)	(28.8-63.0)	(35.9-77.2)
Mauritius*	34.9	37.2	32.6	29.2	38.5	34.4	36.3	42.0
	(29.6-40.2)	(31.0-43.5)	(25.4-39.8)	(23.8-34.6)	(32.3-44.7) *	(29.0-39.8)	(26.7-45.9)	(29.3-54.7)
Mozambique*	25.0	32.5	18.2	19.0	27.8	26.9	19.0	-
	(16.2-33.7)	(23.8-41.1)*	(9.8-26.6)	(10.6-27.3)	(17.2-38.5) *	(18.9-35.0)	(0.0-94.4)	
Namibia*	34.3	37.5	32.4	32.0	35.2	35.8	32.6	32.9
	(30.3-38.4)	(33.0-42.0) *	(27.7-37.1)	(26.0-38.1)	(30.8-39.6)	(31.9-39.7)	(22.7-42.6)	(18.9-46.8)
Seychelles	29.0	28.3	29.5	26.2	31.6	31.4	34.7	24.6
	(26.6-31.3)	(25.0-31.6)	(26.3-32.7)	(23.0-29.4)	(27.8-35.4)	(28.4-34.4)	(26.9-42.5)	(14.2-35.0)
United Republic	28.5	28.0	29.0	24.2	32.2	33.8	-	24.6
of Tanzania*	(25.1-32.0)	(24.2-31.8)	(24.9-33.0)	(19.8-28.5) *	(29.7-36.5)	(30.1-37.4)		(0.00-100)
**Pooled estimates**	**41.3**	**42.8**	**39.9**	**37.1**	**43.9**	**42.6**	**44.1**	**45.3**
	**(28.5-54.1)**	**(27.2-58.5)**	**(29.6-50.1)**	**(26.3-47.8)**	**(31.2-56.6)**	**(30.2-55.0)**	**(31.6-56.7)**	**(31.0-59.6)**
**I^2(%)**	98.8	99.1	96.6	97.1	98.3	98.6	86.8	87.6
**Region of the Americas**								

Antigua and	34.8	32.9	37.1	30.8	37.0	38.8	36.3	32.0
Barbuda*	(29.4-40.1)	(27.3-38.4) *	(30.8-43.5)	(26.9-34.8)	(29.8-44.3)	(29.3-48.4)	(0.0-73.6)	(2.9-61.0)
Argentina*	55.3	56.3	54.2	53.1	56.3	56.9	59.2	57.6
	(52.4-58.1)	(52.9-59.7)	(51.1-57.4)	(48.1-58.2)	(53.5-59.0)	(53.6-60.3)	(54.6-63.9)	(52.6-62.5)
Barbados*	16.2	17.8	14.5	18.2	15.3	16.1	13.1	16.5
	(13.9-18.5)	(14.3-21.3)	(11.9-17.1)	(13.5-22.9)	(12.8-17.8)	(13.5-18.7)	(6.5-19.8)	(6.9-26.2)
Belize*	49.4	52.1	47.2	49.8	49.2	49.3	53.8	54.5
	(41.9-57.0)	(45.4-58.8) *	(38.7-55.7)	(41.4-58.1)	(40.3-58.0)	(42.4-56.2)	(40.5-67.1)	(37.2-71.8)
Bolivia	46.3	43.5	49.8	42.8	48.0	46.3	48.2	46.8
	(41.5-51.0)	(38.6-48.4) *	(44.1-55.6)	(37.4-48.2)	(42.1-53.9)	(41.1-51.5)	(40.9-55.5)	(40.1-53.5)
Chile	50.3	47.4	52.7	49.7	50.6	50.2	44.4	60.8
	(44.8-55.8)	(41.0-53.7)	(46.3-59.2)	(45.1-54.2)	(42.7-58.5)	(44.2-56.1)	(34.8-53.9)	(48.7-72.9)
Bahamas*	24.9	25.1	24.5	22.1	27.8	23.4	29.4	32.1
	(19.5-30.3)	(19.4-30.7)	(18.4-30.5)	(15.6-28.6)	(21.2-34.4)	(18.6-28.3)	(17.6-41.2)	(16.7-47.5)
Costa Rica*	43.3	43.2	43.3	36.2	47.1	42.8	48.0	40.4
	(37.7-48.8)	(37.0-49.4)	(37.1-49.8)	(30.6-41.7) *	(41.0-53.1)	(37.4-48.3)	(40.3-55.6)	(30.7-50.2)
Curaçao*	28.6	31.1	26.9	24.1	30.2	24.7	28.3	44.3
	(24.9-32.3)	(26.7-35.4) *	(21.2-30.6)	(19.7-28.5)	(25.7-34.7)	(20.3-29.0)	(17.4-39.2)	(24.0-64.6)
El Salvador*	44.6	44.2	45.5	47.8	43.3	44.5	42.4	35.7
	(38.3-51.0)	(35.8-52.6)	(39.0-52.0)	(39.7-55.9)	(36.2-50.4)	(38.0-51.0)	(29.1-55.7)	(25.4-46.0)
Guatemala	39.5	38.2	42.0	35.3	42.5	42.2	39.4	26.8
	(35.4-43.7)	(33.5-42.9)	(36.6-47.4)	(28.5-42.2) *	(37.7-47.4)	(35.9-48.4)	(29.1-49.6)	(18.5-35.2)
Guyana*	30.4	32.9	27.9	28.6	30.9	30.5	29.7	27.2
	(24.9-35.9)	(26.3-39.6)	(22.0-33.7)	(21.0-36.3)	(25.0-36.9)	(24.9-36.0)	(20.8-38.6)	(14.2-40.3)
Honduras*	46.1	48.9	44.3	45.8	46.7	44.6	53.8	52.1
	(39.3-52.8)	(41.9-55.9)	(35.5-53.0)	(36.8-54.9)	(40.6-52.8)	(38.2-51.0)	(42.0-65.5)	(39.8-64.4)
Peru	53.8	51.1	56.5	48.8	55.4	55.5	54.2	48.1

	(48.5-59.1)	(45.9-56.3) *	(49.9-63.1)	(39.4-58.1)	(50.4-60.5)	(49.5-61.4)	(44.5-63.0)	(40.8-55.5)
Suriname	46.1	47.0	45.6	40.6	48.7	46.6	48.7	36.3
	(38.4-53.8)	(39.5-54.4)	(37.3-53.9)	(32.7-48.4) *	(40.8-56.5)	(38.9-54.3)	(35.5-61.8)	(25.9-46.7)
Trinidad and	21.7	23.0	20.5	16.4	26.1	21.4	19.3	20.3
Tobago*	(18.1-25.2)	(19.2-26..8)	(15.1-25.8)	(12.7-20.2) *	(21.5-30.7)	(18.1-24.7)	(10.4-28.2)	(10.0-30.6)
Uruguay*	56.2	57.6	55.1	57.4	55.9	56.3	52.6	60.2
	(50.7-61.8)	(51.5-63.6)	(49.0-61.2)	(50.8-64.0)	(50.3-61.5)	(50.3-62.2)	(43.9-61.4)	(51.6-68.8)
**Pooled estimates**	**40.4**	**41.8**	**40.4**	**37.9**	**41.8**	**40.5**	**41.3**	**40.9**
	**(32.9-47.8)**	**(35.1-48.4)**	**(32.4-48.4)**	**(31.3-44.6)**	**(33.8-49.7)**	**(32.8-48.3)**	**(33.5-49.2)**	**(33.8-48.0)**
**I^2(%)**	97.8	96.0	97.4	95.7	97.6	97.6	91.7	88.3
**Eastern Mediterranean Region**								
Afghanistan	55.3	60.5	50.7	49.0	57.3	58.5	53.7	48.7
	(47.7-63.0)	(50.3-70.6) *	(43.9-57.4)	(40.9-57.2)	(49.2-65.5)	(49.2-67.9)	(45.5-61.9)	(37.2-60.1)
Egypt	49.5	45.2	53.2	51.8	46.8	51.1	41.1	47.0
	(42.1-57.0)	(35.3-55.2) *	(43.7-62.6)	(41.9-61.6)	(36.8-57.9)	(42.4-59.8)	(34.9-47.9)	(36.6-57.4)
Iraq*	40.1	48.0	30.8	36.1	42.4	40.6	41.6	33.5
	(34.7-45.5)	(42.4-53.6) *	(22.5-39.2)	(28.7-43.6)	(37.0-47.7)	(35.0-46.3)	(34.4-48.9)	(23.5-43.5)
Kuwait*	16.9	19.3	14.1	16.9	17.0	17.6	14.3	13.7
	(10.6-23.2)	(13.2-25.5) *	(11.2-17.0)	(11.4-22.5)	(10.5-23.5)	(10.3-24.9)	(6.4-22.3)	(5.7-21.7)
Lebanon*	14.9	14.8	15.1	9.5	18.6	13.6	11.8	11.7
	(8.5-21.4)	(7.9-21.7)	(8.2-21.9)	(6.4-12.6) *	(9.2-28.0)	(5.3-21.9)	(4.9-18.7)	(4.0-19.4)
Morocco*	56.2	56.6	56.2	57.1	55.8	55.1	57.8	60.8
	(52.3-60.1)	(53.2-60.0)	(49.4-62.9)	(52.3-61.8)	(51.1-60.4)	(51.1-59.2)	(52.0-63.6)	(55.0-66.5)
Oman*	15.5	19.6	11.7	12.9	16.2	15.6	17.5	15.3
	(12.7-18.3)	(16.6-22.7) *	(9.3-14.1)	(9.1-16.7)	(13.2-19.3)	(12.6-18.7)	(9.8-25.1)	(10.2-20.3)

Pakistan*	49.5	60.3	32.9	41.1	52.0	49.6	47.8	47.7
	(41.9-57.1)	(55.8-64.8)*	(28.0-37.8)	(31.8-50.5) *	(45.1-59.0)	(41.9-57.2)	(37.9-57.7)	(38.3-57.1)
Qatar	36.8	38.5	35.5	37.1	36.3	37.9	27.6	44.0
	(33.3-40.3)	(33.1-43.8)	(31.2-39.8)	(32.0-42.2)	(31.5-41.0)	(32.1-43.8)	(18.0-37.2)	(26.6-61.3)
Sudan*	25.8	28.5	23.0	23.0	26.9	25.9	24.4	31.7
	(21.7-30.0)	(25.3-31.8)	(16.0-30.0)	(16.1-30.0)	(21.9-32.0)	(21.4-30.4)	(16.7-32.1)	(29.4-34.0)
Syrian Arab	41.0	38.9	43.2	37.7	43.9	42.3	38.3	43.6
Republic	(35.2-46.8)	(36.0-41.7)	(34.3-52.1)	(30.4-44.9)	(37.5-50.2)	(36.3-48.3)	(31.3-45.3)	(32.1-55.1)
United Arab	10.4	16.5	6.4	7.4	11.8	10.4	10.2	10.0
Emirates*	(7.3-13.5)	(11.8-21.2) *	(4.8-7.9)	(4.0-10.8) *	(8.5-15.1)	(7.3-13.5)	(6.7-13.8)	(2.8-17.3)
**Pooled estimates**	**34.2**	**37.1**	**30.7**	**31.4**	**35.3**	**34.7**	**32.1**	**33.6**
	**(24.4-44.0)**	**(27.9-46.3)**	**(21.9-39.6)**	**(21.0-41.8)**	**(25.4-45.2)**	**(24.4-45.0)**	**(21.4-42.8)**	**(24.2-43.0)**
**I^2(%)**	98.2	98.0	98.3	97.9	97.7	97.9	96.7	95.6
**Southeast Asia Region**								
Bangladesh	58.1	61.1	53.1	48.5	62.2	57.9	60.4	59.9
	(52.4-63.8)	(53.0-69.1) *	(46.0-60.1)	(40.8-56.3) *	(55.7-68.8)	(52.7-63.1)	(49.3-71.6)	(52.3-67.4)
Indonesia*	34.0	34.6	33.5	34.8	33.3	33.9	33.6	39.6
	(27.8-40.2)	(28.5-40.7)	(27.0-40.0)	(28.5-41.0)	(27.9-38.8)	(27.8-39.9)	(26.6-40.6)	(29.7-49.5)
Thailand	34.2	32.0	36.4	34.5	34.2	34.8	30.9	33.8
	(31.6-36.9)	(28.7-35.2)	(32.2-40.5)	(30.6-38.3)	(31.2-37.2)	(32.2-37.4)	(24.1-37.6)	(24.7-43.0)
Timor-Leste*	15.7	17.2	13.2	14.7	15.7	15.7	15.7	12.0
	(12.9-18.4)	(13.1-21.4) *	(10.8-15.7)	(11.7-17.7)	(13.2-18.2)	(13.0-18.3)	(8.8-22.5)	(6.5-17.5)
**Pooled estimates**	**35.4**	**35.9**	**33.9**	**32.9**	**36.1**	**35.4**	**34.6**	**36.2**
	**(19.7-51.0)**	**(21.9-49.8)**	**(16.4-51.4)**	**(18.6-47.2)**	**(19.9-52.4)**	**(19.4-51.6)**	**(19.6-49.6)**	**(13.3-59.2)**
**I^2(%)**	98.6	96.9	98.3	97.3	98.6	98.7	93.5	97.2
**Western Pacific**								

**Region**								
Brunei	15.1	16.6	13.6	14.4	15.4	15.7	11.3	12.5
Darussalam*	(12.8-17.4)	(13.3-19.9)	(10.7-16.5)	(10.4-18.4)	(12.5-18.3)	(13.1-18.3)	(7.1-15.6)	(5.3-19.7)
Cambodia*	59.7	59.7	60.0	57.6	60.6	59.8	61.1	57.7
	(53.2-66.2)	(51.5-67.9)	(53.4-66.6)	(51.7-63.4)	(53.3-67.9)	(53.1-66.6)	(50.6-71.5)	(40.5-74.8)
Kiribati	39.3	40.0	38.8	34.6	41.1	40.9	35.5	34.5
	(34.9-43.7)	(35.3-44.7)	(33.7-43.8)	(30.6-38.7) *	(35.6-46.5)	(35.7-46.2)	(27.3-43.7)	(22.9-46.1)
Lao People's	38.8	37.6	40.7	47.8	38.0	37.9	43.0	36.9
Democratic	(30.946.7)	(27.8-47.4)	(33.5-47.9)	(36.2-59.5)	(30.4-45.5)	(30.7-45.1)	(31.0-55.1)	(25.4-48.4)
Republic*								
Malaysia*	38.5	39.4	37.7	35.9	39.8	38.9	38.9	37.5
	(35.3-41.7)	(35.9-42.9)	(34.2-41.1)	(32.6-39.1) *	(36.1-43.5)	(35.6-42.2)	(34.1-43.8)	(33.1-42.2)
Mongolia	69.8	65.8	73.7	69.0	70.5	70.5	70.4	61.4
	(65.7-73.9)	(61.9-69.7) *	(68.8-78.5)	(64.5-73.5)	(65.8-75.2)	(66.4-74.6)	(65.3-75.6)	(52.5-70.2)
Philippines	34.1	33.1	35.1	32.6	35.0	36.3	38.4	31.9
	(29.9-38.4)	(28.9-37.3)	(30.2-40.0)	(27.3-37.8)	(30.4-39.5)	(32.4-40.3)	(31.0-45.7)	(21.3-42.6)
Samoa	34.9	34.9	33.6	33.9	35.0	32.5	29.2	34.7
	(32.5-37.2)	(30.3-39.5)	(31.0-36.3)	(28.5-39.4)	(31.8-38.3)	(29.5-35.4)	(19.9-38.5)	(24.9-44.6)
Solomon Islands	33.2	32.1	34.4	31.3	33.0	32.4	41.6	47.2
	(24.0-42.3)	(21.9-42.4)	(23.5-45.3)	(21.6-41.1)	(23.7-43.8)	(20.6-44.2)	(21.1-62.0)	(27.4-67.1)
Tonga	38.7	40.6	37.1	39.4	38.7	39.1	41.7	38.9
	(35.5-41.9)	(35.7-45.5)	(33.3-40.9)	(33.0-45.8)	(35.5-41.8)	(35.7-42.4)	(33.3-50.2)	(29.1-48.6)
Vanuatu	65.2	65.9	65.0	61.8	69.5	59.7	64.4	63.8
	(49.9-80.5)	(50.8-80.9)	(47.7-82.4)	(42.0-81.6)	(55.8-83.2)	(41.1-78.3)	(32.1-96.7)	(46.0-81.5)
Vietnam*	78.8	77.0	80.1	-	78.9	80.0	73.4	71.2
	(74.5-82.8)	(72.7-81.4) *	(75.4-84.7)		(74.9-82.9)	(76.0-84.0)	(64.7-82.2)	(51.7-90.7)
Wallis and Futuna	29.8	28.1	31.3	30.0	29.5	29.9	33.8	22.8

	(26.2-33.4)	(23.1-33.1)	(26.3-36.4)	(23.4-36.7)	(24.3-34.6)	(25.2-34.5)	(21.5-46.1)	(10.3-35.4)
**Pooled estimates**	**44.1**	**43.7**	**44.4**	**40.2**	**44.8**	**43.9**	**44.3**	**41.2**
	**(33.9-54.2)**	**(33.2-54.2)**	**(33.8-55.1)**	**(30.8-49.5)**	**(34.0-55.6)**	**(33.1-54.8)**	**(31.8-56.8)**	**(32.6-49.7)**
**I^2(%)**	98.8	98.3	98.6	97.1	98.6	98.8	96.9	88.8
**Total**								
**Pooled estimates***	**39.5**	**40.6**	**38.1**	**36.8**	**40.7**	**39.8**	**39.6**	**39.6**
	**(34.9-44.0)**	**(36.2-44.9)**	**(33.2-43.0)**	**(32.6-40.9)**	**(36.0-45.4)**	**(35.1-44.5)**	**(34.7-44.4)**	**(35.2-44.0)**
**I^2(%)**	0	0	14.7	0	0	0	0	0

*There was significantly difference between different sex, age, and BMI group (P<0.05).

Table S6. The percentage of at least 5 or more days per week attended physical education classes among young adolescents aged 12-15 years.

	Percentage of physical education classes on 5 or more days per week (95% CI)							
	**Overall**	**Boys, %**	**Girls, %**	**12-13, %**	**14-15, %**	**Normal weight, %**	**Overweight, %**	**Obesity, %**
**Africa Region**								
Algeria*	16.2	17.4	15.5	17.1	15.7	16.6	15.2	16.3
	(13.2-19.2)	(13.4-21.4)	(12.8-18.2)	(14.0-20.1)	(12.2-19.1)	(12.9-20.3)	(9.3-21.0)	(9.5-23.0)
Benin	17.4	18.8	14.9	19.2	16.8	17.6	16.0	21.1
	(13.7-21.1)	(14.4-23.1)	(10.3-19.5)	(12.2-26.2)	(13.8-19.9)	(14.5-20.7)	(8.7-23.3)	(16.8-25.4)
Mauritania*	20.5	23.9	17.1	24.6	19.5	21.1	21.5	18.3
	(17.2-23.8)	(18.7-29.2) *	(12.7-21.4)	(17.9-31.3) *	(15.9-23.0)	(16.2-26.1)	(12.0-31.0)	(6.3-30.4)
Mauritius*	21.3	24.2	18.7	21.9	20.9	20.2	23.9	29.5
	(18.4-24.2)	(21.5-26.8) *	(15.8-21.6)	(19.6-24.2)	(16.8-25.0)	(17.5-22.9)	(16.7-31.0)	(21.2-37.8)
Mozambique*	9.4	11.3	7.2	15.8	7.2	7.9	11.5	-
	(4.1-14.6)	(3.0-19.6) *	(3.1-11.3)	(1.2-30.4) *	(3.7-10.7)	(2.2-13.6)	(0.0-23.2)	
Namibia*	23.7	25.1	22.5	24.2	23.4	22.9	25.3	27.3
	(20.0-27.3)	(19.8-30.3)	(19.4-25.6)	(18.1-30.2)	(19.1-27.7)	(19.4-26.4)	(18.4-32.2)	(15.5-39.1)
Seychelles	25.8	24.0	27.3	27.3	24.3	26.1	29.2	26.7
	(23.2-28.4)	(20.7-27.3) *	(24.2-30.4)	(23.5-31.1)	(20.8-27.9)	(23.3-28.9)	(20.6-37.8)	(16.9-36.6)
United Republic of	24.8	28.2	21.8	22.3	26.9	28.4	-	30.7
Tanzania*	(19.8-29.7)	(21.1-35.4) *	(17.3-26.2)	(18.6-26.1)	(20.5-33.3)	(22.1-34.6)		(0.0-100.0)
**Pooled estimates**	**20.1**	**21.9**	**18.2**	**22.0**	**19.1**	**20.2**	**20.6**	**22.5**
	**(16.8-23.3)**	**(19.1-24.8)**	**(14.1-22.3)**	**(19.3-24.7)**	**(15.0-23.3)**	**(16.6-23.7)**	**(16.1-25.0)**	**(18.6-26.3)**
**I^2(%)**	85.9	68.2	90.8	64.5	89.6	85.9	56.0	26.6
**Region of the Americas**								

Antigua and	21.2	21.6	20.6	24.9	19.3	19.6	-	14.8
Barbuda*	(17.3-25.2)	(16.6-26.5)	(15.9-25.2)	(20.1-29.6) *	(14.6-24.0)	(13.0-26.2)		(0.0-39.2)
Argentina*	20.4	23.0	18.0	20.3	20.4	18.3	17.7	21.3
	(18.7-22.2)	(20.0-26.0) *	(16.7-19.2)	(17.8-22.8)	(18.4-22.4)	(16.7-19.9)	(14.2-21.3)	(12.6-30.0)
Barbados*	29.4	31.8	27.0	33.8	27.9	31.2	28.2	18.7
	(26.4-32.5)	(27.6-36.0)	(23.3-30.7)	(29.3-38.3) *	(24.6-31.3)	(27.6-34.8)	(19.1-37.3)	(7.7-29.7)
Belize*	13.3	15.5	11.3	13.9	12.8	12.9	13.6	11.2
	(10.4-16.2)	(11.0-20.1) *	(9.1-13.6)	(10.0-17.7)	(10.0-15.7)	(9.9-15.9)	(5.7-21.6)	(1.3-21.1)
Bolivia	26.9	28.3	25.1	28.3	25.6	26.8	22.2	24.6
	(24.6-29.2)	(25.4-31.3)	(21.8-28.4)	(23.7-32.9)	(22.4-28.8)	(24.1-29.5)	(16.8-27.6)	(19.1-30.2)
Chile	28.3	26.6	29.6	25.5	30.0	28.4	33.3	15.8
	(24.0-32.6)	(22.6-30.6)	(23.7-35.5)	(22.4-28.6)	(24.4-35.7)	(23.2-33.6)	(25.0-41.5)	(7.6-24.0)
Bahamas*	14.8	17.6	12.5	16.7	12.7	13.9	18.7	14.5
	(11.6-18.0)	(11.9-23.3)	(9.4-15.6)	(13.8-19.6)	(7.9-17.6)	(9.7-18.0)	(14.0-23.4)	(3.3-25.7)
Costa Rica*	31.5	32.4	30.7	34.4	29.9	31.5	33.6	28.5
	(28.5-34.4)	(29.2-35.6)	(26.9-34.5)	(29.2-39.6) *	(27.0-32.7)	(28.9-34.1)	(26.3-40.9)	(18.1-39.0)
Curaçao*	27.7	30.3	25.4	25.9	28.6	28.1	28.2	32.1
	(23.9-31.5)	(25.3-35.3)	(21.1-29.7)	(20.7-31.1)	(24.7-32.4)	(23.8-32.4)	(16.8-39.7)	(14.8-49.4)
El Salvador*	33.7	34.6	32.3	33.4	33.8	32.3	34.2	43.4
	(30.9-36.4)	(30.1-39.0)	(28.8-35.9)	(28.8-38.0)	(30.1-37.5)	(29.0-35.6)	(25.0-43.4)	(30.8-55.9)
Guatemala	28.5	28.2	29.5	29.9	28.3	30.4	32.4	28.1
	(22.3-34.7)	(21.3-35.1)	(22.6-36.4)	(24.5-35.4)	(21.4-35.2)	(23.2-37.7)	(15.4-49.5)	(18.4-37.9)
Guyana*	11.9	12.3	11.4	14.4	10.9	11.9	13.1	9.0
	(9.4-14.4)	(9.3-15.4)	(8.8-13.9)	(11.3-17.4) *	(8.3-13.5)	(9.5-14.3)	(6.5-19.6)	(0.6-17.4)
Honduras*	27.9	30.0	25.8	32.6	23.5	28.3	24.3	27.6
	(24.5-31.3)	(25.8-34.2)	(21.5-30.1)	(27.2-38.0) *	(19.9-27.1)	(24.1-32.5)	(16.5-32.1)	(20.4-34.9)
Peru	1.7	1.7	1.6	1.5	1.7	1.6	0.8	1.5

	(0.0-3.4)	(0.0-3.7)	(0.0-3.4)	(0.0-3.0)	(0.0-3.8)	(0.0-3.4)	(0.0-2.0)	(0.0-3.5)
Suriname	16.1	17.7	14.7	17.1	15.5	15.8	13.3	24.2
	(13.8-18.4)	(15.1-20.3)	(11.1-18.3)	(14.1-20.1)	(12.5-18.4)	(13.3-18.4)	(7.9-18.8)	(14.8-33.6)
Trinidad and	21.3	21.5	21.2	21.2	21.5	20.7	20.5	32.9
Tobago*	(19.2-23.4)	(17.4-25.6)	(18.5-23.9)	(17.6-24.9)	(17.9-25.1)	(18.2-23.3)	(16.1-24.9)	(21.7-44.2)
Uruguay*	27.1	27.0	27.3	29.3	26.3	26.6	26.1	36.7
	(25.0-29.1)	(23.9-30.1)	(25.0-29.5)	(26.1-32.5)	(23.8-28.8)	(24.1-29.1)	(20.0-32.3)	(29.2-44.1)
**Pooled estimates**	**22.4**	**23.5**	**21.3**	**23.6**	**21.6**	**22.2**	**22.1**	**22.5**
	**(17.7-27.1)**	**(18.1-28.9)**	**(16.8-25.7)**	**(18.0-29.2)**	**(16.8-26.4)**	**(17.3-27.0)**	**(15.1-29.2)**	**(14.8-30.1)**
**I^2(%)**	98.1	97.5	97.7	97.9	97.5	98.0	97.0	94.4
**Eastern Mediterranean Region**								
Afghanistan	18.0	16.2	21.4	21.6	17.0	20.4	15.3	34.5
	(13.2-22.7)	(9.2-23.2)	(14.8-27.9)	(12.9-30.3)	(11.9-22.1)	(15.5-25.4)	(4.8-25.8)	(20.0-48.9)
Egypt	13.3	14.8	12.0	14.3	12.7	13.5	10.3	17.9
	(8.9-17.8)	(9.1-20.6)	(5.8-18.2)	(6.6-22.0)	(8.9-16.6)	(7.7-19.3)	(3.6-17.0)	(10.1-25.6)
Iraq*	25.4	30.6	19.2	29.0	23.3	26.2	24.8	21.7
	(22.9-28.0)	(28.3-32.9) *	(15.6-22.7)	(25.6-32.3) *	(19.6-26.9)	(23.2-29.2)	(16.8-32.8)	(11.4-32.0)
Kuwait*	8.0	8.6	7.5	7.9	8.1	8.6	5.0	3.2
	(4.6-11.4)	(3.9-13.2)	(4.6-10.3)	(4.3-11.4)	(4.2-11.9)	(4.6-12.6)	(0.5-9.5)	(0.0-6.4)
Lebanon*	29.0	30.9	27.4	30.5	28.2	29.3	32.8	34.1
	(25.2-32.8)	(26.9-35.0)	(22.1-32.8)	(26.6-34.4)	(22.5-33.8)	(25.4-33.2)	(24.5-41.0)	(18.5-49.6)
Morocco*	26.9	27.1	26.8	27.3	26.6	26.1	33.3	27.0
	(23.9-30.0)	(22.9-31.3)	(23.7-30.0)	(22.0-32.5)	(23.6-29.6)	(24.1-28.1)	(23.3-43.3)	(19.5-34.5)
Oman*	42.7	43.4	42.3	44.6	42.2	44.0	41.0	37.4
	(39.4-46.1)	(40.3-46.4)	(39.4-45.2)	(39.5-49.6)	(38.7-45.7)	(40.4-47.5)	(32.7-49.2)	(29.5-45.4)

Pakistan*	7.9 (6.0-9.8)	9.8 (7.0-12.5) *	5.1 (3.9-6.2)	7.7 (5.8-9.6)	8.0 (5.7-10.2)	8.2 (6.1-10.4)	8.8 (5.6-11.9)	8.1 (4.6-11.6)
Qatar	20.5 (17.3-23.7)	22.2 (18.0-26.4)	19.5 (14.5-24.4)	20.9 (17.2-24.6)	19.9 (15.8-24.1)	22.4 (17.0-27.7)	23.1 (15.0-31.3)	16.8 (8.4-25.1)
Sudan*	8.8 (6.2-11.4)	12.4 (10.0-14.8) *	4.9 (1.9-7.9)	6.7 (2.1-11.2) *	9.7 (6.7-12.7)	8.7 (5.9-11.4)	4.7 (1.7-7.7)	7.4 (3.3-11.4)
Syrian Arab Republic	21.2 (17.1-25.3)	24.6 (21.1-28.1)	17.7 (12.8-22.6)	19.7 (15.1-24.2)	22.5 (18.0-27.1)	21.8 (17.3-26.2)	23.7 (16.1-31.2)	16.1 (9.1-23.1)
United Arab Emirates*	20.4 (17.7-23.1)	21.5 (17.2-25.7)	19.7 (17.6-21.8)	22.1 (19.0-25.1)	19.5 (15.5-23.5)	20.1 (16.8-23.4)	20.8 (12.6-29.1)	23.6 (14.3-32.9)
**Pooled estimates**	**20.2 (14.2-26.2)**	**21.9 (15.4-28.4)**	**18.6 (11.4-25.8)**	**21.0 (14.5-27.5)**	**19.8 (13.63-25.9)**	**20.8 (14.6-27.0)**	**19.8 (13.4-26.3)**	**19.5 (13.4-25.7)**
**I^2(%)**	97.8	97.5	98.6	97.1	97.1	97.6	93.0	91.2
**Southeast Asia Region**								
Bangladesh	27.1 (24.0-30.1)	27.1 (23.8-30.4)	27.1 (23.8-30.4)	26.6 (21.4-31.8)	27.2 (23.3-31.1)	26.1 (22.1-30.2)	30.0 (19.3-40.7)	37.6 (30.9-44.3)
Indonesia*	8.8 (7.8-9.8)	11.1 (9.8-12.4)	6.5 (5.3-7.7)	8.4 (7.4-9.5)	9.1 (7.8-10.5)	8.7 (7.5-9.9)	9.3 (7.0-11.6)	7.9 (4.2-11.6)
Thailand	7.0 (5.8-8.2)	8.6 (6.4-10.9) *	5.5 (4.4-6.6)	8.2 (6.5-9.8) *	6.1 (4.7-7.6)	6.8 (5.6-7.9)	7.9 (2.8-12.9)	9.7 (3.8-15.7)
Timor-Leste*	18.4 (15.7-21.2)	20.3 (16.6-24.1)	17.4 (14.4-20.5)	17.5 (14.4-20.7) *	19.7 (15.9-23.5)	18.1 (15.2-21.0)	17.0 (12.1-21.9)	19.3 (10.8-27.8)
**Pooled estimates**	**15.2 (8.8-21.5)**	**16.6 (9.5-23.8)**	**13.9 (7.1-20.8)**	**14.5 (9.4-19.6)**	**15.3 (8.4-22.1)**	**14.6 (8.9-20.2)**	**14.5 (8.0-20.9)**	**18.5 (5.2-31.8)**
**I^2(%)**	98.4	97.2	98.4	95.9	97.7	97.5	86.0	95.1
**Western Pacific**								

**Region**								
Brunei	20.1	21.5	18.8	23.4	18.6	20.1	20.7	23.0
Darussalam*	(17.7-22.4)	(18.2-24.9) *	(15.6-22.0)	(20.1-26.8) *	(15.7-21.4)	(17.5-22.6)	(14.2-27.2)	(15.8-30.2)
Cambodia*	6.2	7.9	4.4	6.7	5.8	6.1	6.6	3.6
	(4.2-8.1)	(5.3-10.4) *	(1.7-7.2)	(3.1-10.3)	(4.1-7.6)	(3.6-8.7)	(2.1-11.1)	(1.3-5.9)
Kiribati	24.7	25.1	24.4	27.9	23.1	24.9	23.7	14.8
	(21.2-28.1)	(19.9-30.2)	(20.7-28.1)	(25.7-30.2) *	(18.9-27.3)	(20.6-29.2)	(16.3-31.2)	(3.9-25.8)
Lao People's	9.6	11.0	8.1	9.4	9.5	9.3	12.3	8.4
Democratic	(6.9-12.3)	(7.0-14.9)	(4.8-11.4)	(5.8-13.0)	(6.5-12.5)	(6.3-12.4)	(6.2-18.5)	(3.5-13.4)
Republic*								
Malaysia*	21.0	23.4	18.7	23.6	19.7	20.9	21.6	21.8
	(19.8-22.3)	(22.0-24.7) *	(17.1-20.3)	(21.8-25.3) *	(18.3-21.2)	(19.7-22.2)	(19.3-24.0)	(17.9-25.7)
Mongolia	3.7	4.2	3.2	4.0	3.5	3.6	4.5	2.6
	(2.9-4.5)	(3.1-5.3)	(2.3-4.0)	(2.8-5.2)	(2.7-4.3)	(2.7-4.6)	(2.4-6.6)	(0.7-4.5)
Philippines	34.1	32.9	35.2	36.5	32.8	36.7	32.3	27.1
	(30.8-37.4)	(29.5-36.4)	(31.3-39.2)	(32.7-40.3)	(29.2-36.5)	(33.7-39.6)	(26.9-37.8)	(18.7-35.4)
Samoa	14.7	12.2	17.2	12.8	15.4	15.5	14.3	16.0
	(11.3-18.0)	(8.8-15.5) *	(13.3-21.1)	(9.4-16.1) *	(11.6-19.1)	(12.7-18.4)	(8.8-19.8)	(9.0-23.0)
Solomon Islands	28.3	25.7	28.3	23.1	29.2	26.4	35.2	30.0
	(22.2-34.4)	(18.3-33.0)	(21.9-34.7)	(14.8-31.4) *	(23.3-35.1)	(18.7-34.1)	(24.1-46.3)	(20.2-39.7)
Tonga	16.8	16.7	17.0	16.8	16.8	17.3	15.3	12.1
	(14.9-18.8)	(14.0-19.5)	(14.5-19.6)	(12.7-20.9)	(14.6-19.1)	(14.9-19.7)	(9.0-21.6)	(5.2-18.9)
Vanuatu	15.5	16.5	15.1	13.5	17.0	15.5	16.3	5.9
	(8.6-22.4)	(5.4-27.6)	(10.5-19.7)	(7.9-19.0)	(9.4-24.6)	(10.4-20.6)	(0.0-38.8)	(0.0-18.9)
Vietnam*	3.3	2.8	3.8	-	3.4	3.3	1.7	4.2
	(1.8-4.8)	(1.2-4.3)	(1.8-5.8)		(1.9-4.9)	(1.9-4.6)	(0.0-4.2)	(0.0-9.5)
Wallis and Futuna	6.9	8.7	5.3	7.3	6.7	7.1	8.4	4.7

	(4.4-9.5)	(4.1-13.4)	(2.5-8.2)	(3.0-11.6)	(3.8-9.7)	(4.3-9.8)	(0.6-16.1)	(0.0-12.4)
**Pooled estimates**	**15.5**	**16.1**	**14.8**	**16.5**	**15.3**	**15.5**	**15.7**	**14.2**
	**(11.6-19.4)**	**(11.7-20.5)**	**(11.1-18.6)**	**(11.9-21.1)**	**(11.5-19.1)**	**(11.4-19.5)**	**(11.2-20.2)**	**(9.7-18.8)**
**I^2(%)**	98.7	98.3	98.3	98.3	98.3	98.7	94.9	93.4
**Total**								
**Pooled estimates***	**18.8**	**20.2**	**17.5**	**19.6**	**18.2**	**18.7**	**18.4**	**19.4**
	**(16.1-21.5)**	**(17.3-23.2)**	**(14.8-20.2)**	**(16.2-22.9)**	**(15.7-20.7)**	**(15.8-21.6)**	**(15.6-21.1)**	**(15.5-23.3)**
**I^2(%)**	43.3	45.0	33.9	61.8	24.4	47.0	19.4	51.0

*There was significantly difference between different sex, age, and BMI group (P<0.05).

Table S7. The percentage of spent 3 or more hours per day in had no sedentary behavior outside school among young adolescents aged 12-15 years. SB Q52new

	Percentage of 3 or more hours per day in sedentary behavior (95% CI)							
	**Overall**	**Boys, %**	**Girls, %**	**12-13, %**	**14-15, %**	**Normal weight, %**	**Overweight, %**	**Obesity, %**
**Africa Region**								
Algeria*	26.7 (24.3-29.0)	29.6 (27.1-32.2) *	24.1 (20.9-27.4)	21.5 (18.5-24.5) *	31.0 (27.7-34.4)	26.1 (23.4-28.9)	31.6 (26.0-37.2)	32.3 (24.0-40.6)
Benin	18.5 (15.2-21.8)	20.2 (16.4-23.9) *	15.8 (11.3-20.3)	19.4 (14.3-24.5)	18.2 (15.1-21.3)	19.2 (15.9-22.6)	15.0 (6.9-23.2)	22.6 (12.8-32.5)
Mauritania*	39.1 (32.9-45.3)	38.9 (31.7-46.2)	39.4 (31.6-47.2)	37.3 (29.1-45.5)	39.6 (32.0-47.3)	40.4 (33.0-47.7)	41.6 (33.2-50.0)	39.1 (13.3-65.0)
Mauritius*	39.1 (35.4-42.8)	37.8 (33.7-41.9)	40.3 (35.6-45.0)	28.1 (22.9-33.3) *	46.1 (42.8-49.4)	39.3 (35.4-43.2)	36.6 (31.5-41.6)	40.9 (29.6-52.1)
Mozambique*	41.2 (31.8-50.6)	34.4 (26.5-42.4) *	48.4 (37.2-59.7)	39.6 (34.2-45.0)	41.6 (32.0-51.1)	48.8 (37.4-60.2)	44.5 (0.0-94.0)	-
Namibia*	36.9 (34.2-39.6)	35.0 (31.3-38.7)	38.1 (35.6-40.7)	36.2 (30.0-42.5)	37.3 (33.9-40.6)	37.0 (34.6-39.4)	38.2 (31.9-44.6)	46.0 (33.9-58.1)
Seychelles	49.2 (46.3-52.0)	45.2 (41.3-49.0) *	52.7 (49.2-56.3)	44.4 (39.7-49.1) *	53.8 (50.4-57.1)	48.4 (45.0-51.8)	51.9 (43.8-60.1)	50.5 (40.8-60.1)
United Republic of Tanzania*	19.7 (16.7-22.8)	19.4 (15.4-23.4)	20.0 (16.7-23.4)	19.1 (15.8-22.4)	20.2 (16.0-24.4)	21.6 (12.9-30.3)	-	12.2 (0.0-44.1)
**Pooled estimates**	**33.6 (25.6-41.7)**	**32.4 (26.1-38.8)**	**34.6 (25.3-43.8)**	**30.5 (23.4-37.6)**	**35.9 (26.3-45.5)**	**34.9 (27.3-42.4)**	**36.0 (28.0-44.0)**	**35.8 (26.5-45.1)**
**I^2(%)**	97.8	94.9	97.6	94.7	97.9	96.6	86.6	75.4
**Region of the Americas**								

Antigua and	54.4	50.1	59.0	50.2	56.8	65.2	70.2	28.0
Barbuda*	(50.6-58.3)	(45.2-55.0) *	(53.7-64.2)	(45.2-55.1)	(52.4-61.2)	(56.8-73.6)	(31.2-100.0)	(9.4-46.6)
Argentina*	49.9	46.5	53.0	45.1	52.2	51.8	49.5	48.4
	(48.3-51.5)	(43.6-49.4) *	(50.9-55.1)	(42.3-48.0) *	(50.7-53.7)	(50.0-53.7)	(45.5-53.6)	(39.7-57.2)
Barbados*	65.2	60.7	69.7	58.0	67.6	64.4	66.9	73.8
	(62.1-68.3)	(56.1-65.3) *	(66.0-73.4)	(53.5-62.6) *	(64.5-70.7)	(60.8-68.1)	(57.7-76.2)	(63.8-83.8)
Belize*	36.7	33.0	40.4	36.3	37.3	37.1	36.1	42.4
	(32.5-40.9)	(28.1-37.8) *	(35. 4-45.3)	(33.0-39.6)	(30.8-43.8)	(32.7-41.5)	(24.8-47.3)	(33.0-51.8)
Bolivia	24.5	24.9	24.6	23.5	25.0	24.8	24.3	28.9
	(21.5-27.4)	(21.2-28.7)	(20.7-28.4)	(18.6-28.4)	(21.8-28.1)	(21.5-28.1)	(16.5-32.0)	(22.2-35.6)
Chile	52.6	50.5	54.7	51.0	53.6	53.8	55.0	36.8
	(48.2-56.9)	(44.0-57.1)	(50.0-59.4)	(45.9-56.2)	(48.0-59.2)	(48.3-59.2)	(47.1-63.0)	(25.8-47.8)
Bahamas*	54.9	49.8	59.4	52.9	57.2	56.8	49.3	49.5
	(51.4-58.4)	(45.8-53.8) *	(54.9-63.8)	(48.5-57.2)	(52.6-61.7)	(53.4-60.2)	(37.7-60.9)	(38.8-60.1)
Costa Rica*	44.0	40.3	47.6	39.9	46.2	42.8	50.8	48.5
	(40.5-47.6)	(36.5-44.0) *	(43.0-52.2)	(35.3-44.5) *	(42.5-49.9)	(38.7-46.8)	(41.0-60.5)	(42.2-54.7)
Curaçao*	58.6	58.3	58.8	51.3	62.0	57.3	65.3	65.0
	(55.4-61.8)	(54.3-62.3)	(54.6-62.9)	(46.3-56.2) *	(58.4-65.6)	(52.3-62.3)	(54.7-75.9)	(47.2-82.8)
El Salvador*	35.0	33.1	37.5	25.7	39.5	35.4	36.6	47.3
	(30.1-40.0)	(27.5-38.6) *	(31.1-43.8)	(19.2-32.2) *	(34.7-44.2)	(29.5-41.2)	(28.4-44.9)	(40.6-53.9)
Guatemala	22.8	21.2	24.8	19.6	24.9	22.0	22.9	26.7
	(16.9-28.7)	(14.6-27.9)	(19.0-30.6)	(13.1-26.1)	(18.2-31.7)	(16.0-28.0)	(16.6-29.3)	(19.1-34.3)
Guyana*	35.7	35.4	35.8	32.4	37.0	34.6	39.9	38.5
	(30.2-41.1)	(30.0-40.8)	(29.3-42.3)	(24.5-40.4)	(31.3-42.8)	(29.4-39.9)	(29.5-50.3)	(30.4-46.7)
Honduras*	30.3	30.0	30.7	29.7	30.8	30.1	34.3	36.4
	(27.7-32.8)	(26.9-33.1)	(26.9-34.5)	(26.4-33.1)	(27.6-34.1)	(27.4-32.7)	(29.3-39.4)	(25.0-47.8)
Peru	28.8	28.3	29.4	25.7	29.8	29.0	29.4	32.5

	(25.2-32.4)	(24.8-31.8)	(24.1-34.8)	(20.8-30.7)	(26.0-33.6)	(25.3-32.7)	(21.6-37.3)	(21.5-43.6)
Suriname	40.7	40.4	40.8	33.5	44.3	41.1	39.1	39.7
	(35.8-45.6)	(34.1-46.8)	(36.2-45.5)	(26.8-40.1) *	(38.1-50.6)	(36.0-46.2)	(32.1-46.2)	(20.1-59.4)
Trinidad and	44.2	39.9	48.2	36.4	50.4	44.4	49.0	44.5
Tobago*	(40.7-47.7)	(32.9-46.8) *	(45.1-51.3)	(33.1-39.7) *	(44.6-56.3)	(39.7-49.1)	(40.4-57.6)	(33.7-55.4)
Uruguay*	58.3	56.5	60.0	53.9	59.9	59.2	60.7	53.3
	(55.1-61.4)	(52.7-60.4) *	(56.0-64.1)	(48.1-59.6) *	(57.1-62.8)	(55.8-62.7)	(53.8-67.5)	(42.8-63.8)
**Pooled estimates**	**43.4**	**41.1**	**45.6**	**39.2**	**45.6**	**44.1**	**44.8**	**43.5**
	**(37.3-49.5)**	**(35.4-46.9)**	**(39.3-51.9)**	**(33.9-44.5)**	**(39.2-52.0)**	**(37.6-50.5)**	**(38.3-51.2)**	**(37.7-49.3)**
**I^2(%)**	98.2	96.7	97.5	95.6	98.1	97.9	91.3	83.7
**Eastern Mediterranean Region**								
Afghanistan	23.6	20.8	27.1	21.3	25.0	23.3	23.0	26.7
	(16.6-30.6)	(12.4-29.2) *	(18.7-35.6)	(15.6-27.0)	(17.2-32.8)	(14.8-31.8)	(9.5-36.6)	(17.7-35.6)
Egypt	27.4	35.5	19.1	26.8	27.8	26.9	27.2	27.5
	(21.5-33.2)	(25.5-43.5) *	(14.0-24.1)	(20.1-33.5)	(21.5-34.1)	(19.8-33.9)	(19.4-34.9)	(18.9-36.2)
Iraq*	26.1	27.8	24.1	25.6	26.3	25.7	30.1	34.4
	(22.2-30.0)	(24.5-31.1)	(19.7-28.5)	(19.3-31.9)	(22.4-30.1)	(21.6-29.8)	(19.1-41.2)	(22.5-46.3)
Kuwait*	62.8	61.3	64.3	59.8	64.3	61.4	66.8	69.3
	(57.0-68.5)	(54.2-68.3)	(60.2-68.4)	(53.8-65.7)	(57.4-71.1)	(55.4-67.4)	(54.9-78.7)	(62.0-76.6)
Lebanon*	46.9	44.9	48.8	44.8	48.4	48.5	49.0	41.6
	(42.4-51.5)	(39.0-50.7)	(44.5-53.1)	(41.7-48.0)	(42.5-54.3)	(43.6-53.5)	(40.3-57.7)	(19.0-64.1)
Morocco*	25.6	25.1	26.0	22.2	28.2	24.9	32.9	27.3
	(22.8-28.4)	(22.1-28.2)	(22.3-29.8)	(18.9-25.5) *	(25.0-31.4)	(21.5-28.4)	(24.1-41.7)	(16.6-38.0)
Oman*	37.8	39.2	36.3	35.2	38.6	37.4	45.5	39.7
	(34.0-41.6)	(35.4-43.0)	(32.6-40.1)	(27.8-42.6)	(34.5-42.8)	(33.2-41.6)	(36.2-54.8)	(31.8-47.6)

Pakistan*	8.3	9.1	6.9	6.2	8.9	8.5	7.9	9.3
	(6.6-9.9)	(8.1-10.1)	(3.8-10.1)	(3.3-9.1)	(7.3-10.5)	(7.0-10.0)	(3.9-12.0)	(5.5-13.0)
Qatar	48.9	42.4	54.6	48.6	49.5	50.2	56.1	67.5
	(45.1-52.7)	(37.8-46.9) *	(49.2-59.9)	(44.1-53.1)	(44.1-54.9)	(44.2-56.3)	(43.2-69.0)	(52.9-82.0)
Sudan*	19.5	22.0	17.0	16.9	19.9	20.1	14.2	16.1
	(15.4-23.6)	(18.5-25.4)	(12.7-21.4)	(10.0-23.7)	(14.9-25.0)	(15.3-24.9)	(8.0-20.3)	(10.8-21.4)
Syrian Arab	25.2	23.6	27.0	23.5	26.7	25.0	28.6	31.9
Republic	(19.9-30.5)	(17.6-29.5)	(19.2-34.7)	(16.2-30.7)	(20.5-32.8)	(19.6-30.4)	(19.2-38.1)	(18.8-44.9)
United Arab	51.2	44.7	55.4	48.4	52.4	51.4	44.0	53.0
Emirates*	(47.2-55.2)	(41.0-48.5) *	(52.0-58.9)	(43.1-53.7)	(48.2-56.6)	(47.5-55.4)	(36.9-51.2)	(43.7-62.3)
**Pooled estimates**	**33.6**	**33.0**	**33.9**	**31.6**	**34.6**	**33.6**	**35.1**	**36.7**
	**(23.3-43.9)**	**(23.2-42.7)**	**(22.7-45.1)**	**(21.4-41.8)**	**(24.1-45.1)**	**(22.7-44.4)**	**(24.5-45.8)**	**(24.33-49.1)**
**I^2(%)**	98.9	98.9	98.8	98.2	98.7	98.8	95.2	96.4
**Southeast Asia**								
**Region**								
Bangladesh	15.1	16.9	11.8	15.1	15.1	15.9	23.2	12.7
	(11.1-19.1)	(10.8-22.9)	(9.0-14.6)	(10.1-20.1)	(12.0-18.2)	(11.7-20.2)	(12.1-34.3)	(6.9-18.5)
Indonesia*	24.4	24.9	23.9	20.7	28.4	24.0	28.6	31.7
	(22.6-26.3)	(22.6-27.1)	(21.8-26.1)	(18.6-22.8) *	(25.9-31.0)	(22.2-25.9)	(24.2-33.1)	(25.9-37.5)
Thailand	50.9	50.4	51.3	43.3	56.9	50.9	52.3	58.3
	(48.0-53.9)	(45.7-55.1)	(47.3-55.3)	(40.1-46.5) *	(53.0-60.8)	(47.5-54.3)	(44.6-60.1)	(48.5-68.1)
Timor-Leste*	15.3	15.8	15.0	13.6	16.0	14.8	17.0	21.6
	(12.6-17.9)	(12.6-19.0)	(11.3-18.6)	(10.9-16.3)	(13.1-18.9)	(11.6-18.0)	(10.3-23.8)	(6.9-36.3)
**Pooled estimates**	**26.4**	**27.0**	**25.5**	**23.2**	**29.1**	**26.4**	**30.3**	**31.1**
	**(11.5-41.4)**	**(14.1-39.9)**	**(11.1-39.8)**	**(10.6-35.8)**	**(12.9-45.2)**	**(11.9-40.9)**	**(16.4-44.3)**	**(12.6-49.5)**
**I^2(%)**	99.2	98.0	98.9	98.6	99.1	98.9	93.7	95.4
**Western Pacific**								

**Region**								
Brunei	54.6	51.0	57.8	45.4	58.7	54.7	51.9	58.7
Darussalam*	(51.3-57.8)	(46.5-55.6)	(53.9-61.7)	(41.1-49.6) *	(54.5-62.8)	(51.6-57.9)	(42.5-61.2)	(47.2-70.2)
Cambodia*	10.2	11.1	9.4	9.4	10.5	10.1	11.9	11.1
	(7.4-12.9)	(8.0-14.2)	(6.1-12.7)	(6.9-11.9)	(6.9-14.1)	(6.6-13.7)	(7.0-16.7)	(6.9-15.2)
Kiribati	14.6	16.0	13.6	14.9	14.2	14.0	15.3	19.6
	(12.3-17.0)	(11.4-20.5)	(11.7-15.6)	(11.2-18.6)	(11.1-17.4)	(11.4-16.6)	(9.7-20.9)	(7.3-32.0)
Lao People's	19.1	19.1	19.1	20.1	19.0	18.8	17.5	28.6
Democratic	(16.4-21.8)	(15.9-22.3)	(15.6-22.7)	(15.6-24.6)	(16.0-21.9)	(16.2-21.4)	(11.9-23.1)	(15.9-41.2)
Republic*								
Malaysia*	42.6	41.8	43.5	35.5	46.3	43.1	43.8	40.2
	(40.9-44.4)	(39.5-44.0)	(41.6-45.5)	(33.4-37.7) *	(44.2-48.5)	(41.2-44.9)	(40.7-46.9)	(36.4-43.9)
Mongolia	40.0	37.1	42.7	33.3	45.3	40.0	43.5	42.0
	(36.2-43.7)	(33.2-41.0)	(38.3-47.2)	(28.8-37.8) *	(41.2-49.3)	(36.1-44.0)	(37.1-49.9)	(35.9-48.1)
Philippines	30.6	30.1	31.1	28.8	31.6	30.3	38.5	40.6
	(26.7-34.4)	(24.7-35.5)	(26.8-35.4)	(24.6-33.1)	(27.4-35.8)	(26.2-34.4)	(32.4-44.6)	(34.0-47.1)
Samoa	37.5	43.3	30.6	36.3	37.8	34.3	34.6	40.4
	(33.6-41.5)	(38.9-47.7) *	(26.8-34.3)	(30.5-42.1)	(33.1-42.4)	(29.7-38.9)	(27.5-41.8)	(29.4-51.4)
Solomon Islands	26.4	26.4	27.0	30.2	24.1	24.3	25.7	31.4
	(20.9-32.0)	(16.8-36.0)	(21.1-32.9)	(25.4-35.1) *	(18.1-30.1)	(18.5-30.1)	(17.2-34.3)	(22.6-40.1)
Tonga	29.2	28.2	30.1	26.6	30.1	27.8	31.3	37.9
	(26.5-32.0)	(24.6-31.8)	(26.3-33.9)	(20.7-32.5)	(27.3-32.8)	(24.5-31.0)	(23.8-38.9)	(27.4-48.3)
Vanuatu	19.1	22.3	16.0	16.6	22.1	18.8	28.3	16.4
	(14.6-23.6)	(15.4-29.2)	(10.6-21.4)	(10.7-22.5) *	(16.7-27.5)	(13.9-23.7)	(7.4-49.2)	(0.0-33.3)
Vietnam*	35.1	33.8	36.2	-	35.1	34.6	39.5	37.2
	(30.6-39.6)	(29.8-37.7)	(30.7-41.8)		(30.7-39.5)	(29.9-39.4)	(31.7-47.3)	(29.6-44.8)
Wallis and Futuna	34.0	28.1	39.2	29.4	37.3	35.1	26.8	33.9

	(29.3-38.7)	(22.4-33.9) *	(33.0-45.5)	(22.0-36.8) *	(32.2-42.4)	(29.9-40.4)	(11.5-42.1)	(21.7-46.2)
**Pooled estimates**	**30.2**	**29.9**	**30.5**	**27.2**	**31.7**	**29.7**	**31.5**	**33.9**
	**(22.6-37.9)**	**(23.0-36.9)**	**(22.0-39.0)**	**(20.2-34.2)**	**(23.8-39.6)**	**(21.96-37.5)**	**(23.7-39.3)**	**(26.0-41.8)**
**I^2(%)**	98.7	97.4	98.7	97.3	98.4	98.5	94.6	92.8
**Total**								
**Pooled estimates***	**34.6**	**33.6**	**35.0**	**31.3**	**36.5**	**34.8**	**36.5**	**38.0**
	**(28.4-40.7)**	**(28.5-38.7)**	**(27.6-42.4)**	**(25.5-37.0)**	**(30.0-43.0)**	**(28.4-41.2)**	**(30.7-42.2)**	**(33.3-42.7)**
**I^2(%)**	60.0	53.2	67.8	62.8	58.4	62.0	53.2	22.7

*There was significantly difference between different sex, age, and BMI group (P<0.05).

Table S8. The percentage of all four active among young adolescents aged 12-15 years.

	Overall	Boys, %	Girls, %	12-13, %	14-15, %	Normal weight, %	Overweight, %	Obesity, %
**Africa Region**								
Algeria*	0.8 (0.3-1.2)	1.1 (0.4-1.8)*	0.6 (0.1-1.0)	0.8 (0.3-1.3)	0.8 (0.3-1.3)	0.7 (0.3-1.1)	1.6 (0.0-3.2)	0.2 (0.2-0.3)
Benin	1.7 (0.6-2.6)	2.2 (1.1-3.3)*	0.6 (0.0-1.6)	2.3 (1.0-3.5)	1.5 (0.4-2.6)	1.7 (0.8-2.6)	0.0 (0.0-0.0)	5.4 (3.3-7.6)
Mauritania*	1.8 (0.3-3.3)	2.3 (0.3-4.4)*	1.3 (0.1-2.5)	1.0 (0.0-2.6)	2.1 (0.3-3.9)	1.9 (0.1-3.7)	0.0 (0.0-0.0)	4.4 (0.0-11.1)
Mauritius*	1.0 (0.4-1.6)	1.6 (0.6-2.6)*	0.4 (0.1-0.8)	0.7 (0.0-1.5)	1.2 (0.5-1.9)	0.9 (0.3-1.6)	1.4 (0.0-3.2)	2.6 (0.0-5.9)
Mozambique*	0.5 (0.01-1.1)	0.5 (0.4-0.7)	0.6 (0.0-1.7)	0.0 (0.0-0.0)	0.7 (0.0-1.5)	-	-	-
Namibia*	1.2 (0.6-1.7)	1.8 (0.8-2.7)*	0.7 (0.2-1.3)	0.6 (0.0-1.2)	1.4 (0.8-2.1)	1.2 (0.6-1.7)	1.2 (0.0-3.0)	1.9 (1.2-2.5)
Seychelles	1.2 (0.6-1.8)	0.8 (0.1-1.6)*	1.6 (0.8-2.4)	1.4 (0.6-2.2)	1.1 (0.2-2.0)	1.6 (0.8-2.4)	0.0 (0.0-0.0)	0.0 (0.0-0.0)
United Republic of Tanzania*	1.3 (0.8-2.0)	1.6 (0.6-2.5)	1.3 (0.5-2.1)	1.6 (1.0-2.2)	1.3 (0.5-2.0)	1.1 (0.0-3.0)	0.0 (0.0-0.0)	0.0 (0.0-0.0)
**Pooled estimates**	**1.0 (0.8-1.3)**	**1.3 (0.8-1.9)***	**0.8 (0.5-1.1)**	**1.1 (0.7-1.5)**	**1.1 (0.8-1.4)**	**1.1 (0.8-1.4)**	**1.4 (0.5-2.3)**	**2.4 (0.7-4)**
**I^2(%)**	20.7	75.5	36.8	49.0	0	26.0	0	92.5
**Region of the Americas**								

Antigua and	1.9	2.1	1.5	2.1	1.9	1.0	0.0	0.0
Barbuda*	(1.1-2.8)	(0.9-3.3)*	(0.5-2.5)	(1.0-3.1)	(0.8-3.0)	(0.0-2.4)	(0.0-0.0)	(0.0-0.0)
Argentina*	1.4	1.6	1.3	1.2	1.5	1.3	1.8	2.1
	(1.0-1.8)	(1.2-1.9)	(0.7-1.9)	(0.7-1.8)	(1.1-1.9)	(1.0-1.6)	(1.1-2.5)	(0.0-4.4)
Barbados*	1.0	1.5	0.5	1.0	1.0	1.1	0.8	0.0
	(0.4-1.6)	(0.4-2.6)*	(0.0-1.0)	(0.0-2.2)	(0.3-1.7)	(0.4-1.9)	(0.7-0.9)	(0.0-0.0)
Belize*	0.9	1.1	0.8	1.1	0.8	1.1	0.6	0.0
	(0.5-1.4)	(0.3-1.8)	(0.1-1.5)	(0.3-1.9)	(0.3-1.4)	(0.5-1.8)	(0.0-1.8)	(0.0-0.0)
Bolivia	0.7	0.9	0.5	0.5	0.8	0.8	0.0	1.2
	(0.4-1.0)	(0.2-1.6)	(0.2-0.8)	(0.0-1.0)	(0.4-1.2)	(0.4-1.2)	(0.0-0.0)	(0.0-2.6)
Chile	1.6	2.2	1.2	1.6	1.7	1.8	0.6	0.0
	(1.0-2.3)	(0.8-3.5)*	(0.3-2.1)	(0.7-2.5)	(0.8-2.6)	(0.9-2.6)	(0.0-2.0)	(0.0-0.0)
Bahamas*	0.7	1.3	0.3	0.7	0.8	1.0	0.0	0.0
	(0.2-1.3)	(0.2-2.4)*	(0.0-0.6)	(0.0-1.3)	(0.0-1.6)	(0.3-1.8)	(0.0-0.0)	(0.0-0.0)
Costa Rica*	1.7	1.9	1.5	2.0	1.5	1.8	2.0	0.9
	(1.1-2.3)	(1.3-2.6)	(0.6-2.3)	(1.0-3.0)	(0.9-2.2)	(1.1-2.6)	(0.7-3.3)	(0.0-2.9)
Curaçao*	1.8	2.4	1.3	1.4	2.0	1.7	1.5	6.0
	(1.1-2.6)	(1.4-3.5)*	(0.3-2.4)	(0.4-2.5)	(1.1-3.0)	(0.7-2.8)	(0.0-4.7)	(0.0-15.1)
El Salvador*	0.7	1.0	0.5	0.3	0.9	0.9	0.5	0.0
	(0.3-1.2)	(0.3-1.7)*	(0.0-1.0)	(0.0-0.8)	(0.3-1.5)	(0.3-1.5)	(0.0-1.4)	(0.0-0.0)
Guatemala	0.8	0.5	1.1	0.1	1.2	0.8	1.6	0.0
	(0.3-1.2)	(0.0-1.0)*	(0.1-2.1)	(0.0-0.1)	(0.4-1.9)	(0.2-1.4)	(0.0-3.9)	(0.0-0.2)
Guyana*	0.5	0.9	0.2	0.4	0.6	0.5	0.0	1.2
	(0.2-0.8)	(0.3-1.4)*	(0.0-0.4)	(0.0-1.0)	(0.2-0.9)	(0.2-0.8)	(0.0-0.0)	(0.0-3.8)
Honduras*	0.9	1.1	0.8	1.3	0.6	1.1	0.7	0.0
	(0.4-1.5)	(0.1-2.0)	(0.0-1.8)	(0.4-2.2)	(0.0-1.3)	(0.4-1.9)	(0.0-2.1)	(0.0-0.0)
Peru	0.1	0.0	0.1	0.0	0.1	0.0	0.0	0.0

	(0.0-0.2)	(0.0-0.0)	(0.0-0.4)	(0.0-0.0)	(0.0-0.3)	(0.0-0.0)	(0.0-0.0)	(0.0-0.0)
Suriname	1.6	2.4	0.9	1.6	1.6	1.2	2.3	2.1
	(0.7-2.5)	(1.0-3.7)*	(0.0-2.0)	(0.4-2.8)	(0.4-2.8)	(0.1-2.3)	(0.0-5.7)	(0.0-7.0)
Trinidad and Tobago*	0.8	1.3	0.4	0.5	1.1	1.1	0.0	0.0
	(0.3-1.4)	(0.5-2.1)*	(0.0-1.0)	(0.0-1.1)	(0.4-1.9)	(0.5-1.8)	(0.0-0.0)	(0.0-0.0)
Uruguay*	2.4	3.2	1.7	3.1	2.1	2.4	2.4	4.7
	(1.6-3.2)	(1.9-4.4)*	(1.0-2.3)	(1.8-4.4)	(1.2-2.9)	(1.5-3.3)	(0.3-4.5)	(0.6-8.9)
**Pooled estimates**	**1.1**	**1.4**	**0.7**	**1.0**	**1.1**	**1.1**	**1.0**	**1.2**
	**(0.8-1.4)**	**(1.1-1.8)***	**(0.5-1.0)**	**(0.7-1.4)**	**(0.8-1.5)**	**(0.9-1.4)**	**(0.7-1.4)**	**(0.2-2.2)**
**I^2(%)**	90.5	61.8	73.1	85.9	85.7	57.2	38.5	61.7
**Eastern Mediterranean Region**								
Afghanistan	0.4	0.3	0.6	0.9	0.3	0.2	0.0	3.5
	(0.01-0.9)	(0.0-0.8)	(0.0-1.5)	(0.0-2.1)	(0.0-0.7)	(0.0-0.5)	(0.0-0.0)	(0.0-7.4)
Egypt	0.6	1.1	0.1	0.5	0.7	0.6	0.0	0.0
	(0.01-1.2)	(0.1-2.0)*	(0.0-0.3)	(0.0-1.4)	(0.0-1.3)	(0.0-1.3)	(0.0-0.0)	(0.0-0.0)
Iraq*	1.3	1.9	0.6	1.3	1.4	1.4	1.5	1.1
	(0.8-1.9)	(1.1-2.8)*	(0.2-1.1)	(0.1-2.4)	(0.7-2.1)	(0.7-2.0)	(0.0-3.7)	(0.0-3.5)
Kuwait*	0.5	0.5	0.5	0.9	0.3	0.7	0.0	0.0
	(0.2-0.7)	(0.0-1.0)	(0.0-1.2)	(0.2-1.5)	(0.0-0.7)	(0.3-1.0)	(0.0-0.0)	(0.0-0.0)
Lebanon*	0.8	1.0	0.5	1.0	0.6	1.0	0.6	0.0
	(0.3-1.2)	(0.4-1.7)*	(0.1-0.9)	(0.3-1.8)	(0.0-1.1)	(0.4-1.5)	(0.0-1.8)	(0.0-0.0)
Morocco*	1.5	1.4	1.7	1.3	1.8	1.7	2.4	0.0
	(0.9-2.1)	(0.7-2.1)	(0.7-2.7)	(0.2-2.3)	(1.2-2.3)	(1.0-2.4)	(0.7-4.1)	(0.0-0.0)
Oman*	0.9	1.5	0.5	1.0	1.0	0.9	1.4	1.1
	(0.5-1.4)	(1.0-1.9)*	(0.1-1.0)	(0.5-1.5)	(0.5-1.4)	(0.3-1.5)	(1.3-1.6)	(0.8-1.3)

Pakistan*	0.1 (0.0-0.2)	0.1 (0.0-0.2)	0.1 (0.0-0.3)	0.0 (0.0-0.0)	0.1 (0.0-0.3)	0.1 (0.0-0.3)	0.0 (0.0-0.0)	0.0 (0.0-0.0)
Qatar	1.7 (1.1-2.3)	2.5 (1.5-3.6)*	1.1 (0.5-1.7)	1.9 (1.1-2.6)	1.6 (0.5-2.7)	2.3 (1.0-3.7)	1.3 (1.1-1.4)	0.1 (0.0-0.4)
Sudan*	0.1 (0.0-0.3)	0.2 (0.1-0.2)	0.1 (0.0-0.2)	0.0 (0.0-0.0)	0.2 (0.0-0.4)	0.6 (0.3-0.9)	1.3 (0.0-2.7)	0.0 (0.0-0.0)
Syrian Arab Republic	0.6 (0.3-1.0)	0.7 (0.2-1.3)	0.5 (0.1-1.0)	0.3 (0.0-0.6)	1.0 (0.3-1.7)	0.3 (0.0-0.6)	0.5 (0.0-1.5)	0.0 (0.0-0.0)
United Arab Emirates*	0.3 (0.0-0.5)	0.6 (0.3-0.8)*	0.1 (0.0-0.2)	0.0 (0.0-0.0)	0.4 (0.0-0.8)	-	-	-
**Pooled estimates**	**0.7 (0.4-0.9)**	**0.8 (0.5-1.0)***	**0.3 (0.2-0.4)**	**0.9 (0.6-1.3)**	**0.7 (0.4-1.0)**	**0.7 (0.4-1.0)**	**1.3 (1.1-1.5)**	**0.8 (-0.1-1.7)**
**I^2(%)**	87.6	89.3	69.2	63.2	83.5	83.3	38.6	92.6
**Southeast Asia Region**								
Bangladesh	1.0 (0.4-1.6)	1.2 (0.4-2.0)*	0.7 (0.3-1.1)	0.9 (0.2-1.6)	1.1 (0.4-1.8)	1.3 (0.5-2.0)	1.0 (0.0-2.9)	0.2 (0.2-0.3)
Indonesia*	0.3 (0.1-0.4)	0.4 (0.2-0.6)	0.1 (0.0-0.3)	0.2 (0.0-0.4)	0.3 (0.0-0.6)	0.3 (0.1-0.5)	0.3 (0.0-0.7)	0.4 (0.0-1.1)
Thailand	0.3 (0.0-0.6)	0.5 (0.0-1.1)	0.1 (0.0-0.4)	0.6 (0.0-1.1)	0.2 (0.0-0.3)	0.3 (0.0-0.6)	0.0 (0.0-0.0)	1.4 (1.0-1.8)
Timor-Leste*	0.4 (0.0-0.8)	0.3 (0.0-0.6)	0.4 (0.0-0.8)	0.2 (0.1-0.2)	0.4 (0.0-0.7)	0.3 (0.0-0.7)	1.4 (0.0-3.3)	0.0 (0.0-0.0)
**Pooled estimates**	**0.4 (0.2-0.6)**	**0.4 (0.2-0.7)**	**0.3 (0.03-0.5)**	**0.3 (0.1-0.4)**	**0.4 (0.1-0.6)**	**0.4 (0.1-0.6)**	**0.5 (-0.04-1.0)**	**0.7 (-0.1-1.4)**
**I^2(%)**	40.9	31.4	67.8	48.3	55.9	54.3	15.6	94.6
**Western Pacific**								

**Region**								
Brunei	0.3	0.4	0.2	0.5	0.2	0.2	0.0	0.0
Darussalam*	(0.0-0.5)	(0.0-0.8)	(0.0-0.5)	(0.0-1.1)	(0.0-0.4)	(0.0-0.7)	(0.0-0.0)	(0.0-0.0)
Cambodia*	0.02	0.0	0.0	0.0	0.0	0.0	0.0	0.0
	(0.0-0.1)	(0.0-0.0)	(0.0-0.1)	(0.0-0.0)	(0.0-0.1)	(0.0-0.1)	(0.0-0.0)	(0.0-0.0)
Kiribati	1.4	1.9	1.0	0.5	1.6	1.4	0.8	0.0
	(0.8-1.9)	(0.5-3.3)	(0.2-1.7)	(0.0-1.1)	(1.0-2.2)	(0.8-2.1)	(0.0-2.6)	(0.0-0.0)
Lao People's	0.2	0.4	0.1	0.5	0.2	0.3	0.0	1.0
Democratic	(0.0-0.5)	(0.0-1.0)	(0.0-0.3)	(0.3-0.6)	(0.0-0.5)	(0.0-0.6)	(0.0-0.0)	(0.0-3.1)
Republic*								
Malaysia*	1.4	1.8	1.0	1.2	1.5	1.4	1.2	0.9
	(1.1-1.6)	(1.5-2.2)*	(0.7-1.2)	(0.8-1.6)	(1.2-1.8)	(1.2-1.7)	(0.5-1.9)	(0.3-1.6)
Mongolia	0.3	0.4	0.3	0.3	0.3	0.4	0.0	0.0
	(0.2-0.5)	(0.1-0.8)	(0.0-0.5)	(0.1-0.6)	(0.1-0.6)	(0.1-0.6)	(0.0-0.0)	(0.0-0.0)
Philippines	0.9	1.1	0.8	0.6	1.1	0.8	1.8	1.3
	(0.5-1.3)	(0.6-1.5)	(0.2-1.3)	(0.2-0.9)	(0.5-1.7)	(0.3-1.2)	(0.2-3.3)	(0.0-3.3)
Samoa	1.3	1.2	1.5	1.9	1.2	1.4	0.7	1.4
	(0.7-2.0)	(0.4-2.1)	(0.3-2.7)	(0.3-3.4)	(0.8-1.6)	(0.7-2.0)	(0.0-2.0)	(0.0-4.5)
Solomon Islands	1.7	2.0	1.5	2.4	1.1	1.6	1.1	0.0
	(0.0-3.8)	(0.0-4.8)	(0.0-3.2)	(0.0-5.6)	(0.0-2.2)	(0.0-3.9)	(0.0-3.5)	(0.0-0.0)
Tonga	1.0	1.0	1.1	1.0	1.1	0.9	1.4	2.2
	(0.5-1.6)	(0.2-1.8)	(0.3-1.8)	(0.2-1.8)	(0.4-1.7)	(0.3-1.5)	(0.0-3.3)	(0.0-5.0)
Vanuatu	0.4	0.3	0.5	0.0	0.8	0.2	0.9	0.0
	(0.0-0.9)	(0.0-0.7)	(0.0-1.4)	(0.0-0.0)	(0.0-1.9)	(0.0-0.6)	(0.0-3.4)	(0.0-0.0)
Vietnam*	0.3	0.5	0.1	-	0.3	0.3	0.0	0.0
	(0.0-0.6)	(0.0-1.2)	(0.0-0.4)		(0.0-0.6)	(0.0-0.6)	(0.0-0.0)	(0.0-0.0)
Wallis and Futuna	0.3	0.6	0.0	0.7	0.0	0.4	0.0	0.0

	(0.0-0.7)	(0.0-1.4)	(0.0-0.0)	(0.0-1.7)	(0.0-0.0)	(0.0-0.9)	(0.0-0.0)	(0.0-0.0)
**Pooled estimates**	**0.6**	**0.8**	**0.5**	**0.6**	**0.7**	**0.6**	**1.1**	**1.0**
	**(0.4-0.9)**	**(0.5-1.2)***	**(0.2-0.7)**	**(0.4-0.9)**	**(0.4-1.0)**	**(0.3-0.9)**	**(0.6-1.5)**	**(0.5-1.6)**
**I^2(%)**	93.6	81.0	88.4	56.0	94.0	93.3	0	0
**Total**								
**Pooled estimates***	**0.7**	**0.9**	**0.5**	**0.8**	**0.8**	**0.8**	**1.1**	**1.0**
	**(0.5-1.0)**	**(0.6-1.3)***	**(0.3-0.7)**	**(0.4-1.1)**	**(0.5-1.1)**	**(0.5-1.14)**	**(0.8-1.3)**	**(0.6-1.3)**
**I^2(%)**	81.5	83.9	76.2	85.9	78.3	81.0	61.0	0

*There was significantly difference in the prevalence of all four active between different sex, age, and BMI group (*P*<0.05).
